# Multicopy suppressor screens reveal convergent evolution of single-gene lysis proteins

**DOI:** 10.1038/s41589-023-01269-7

**Published:** 2023-02-20

**Authors:** Benjamin A. Adler, Karthik Chamakura, Heloise Carion, Jonathan Krog, Adam M. Deutschbauer, Ry Young, Vivek K. Mutalik, Adam P. Arkin

**Affiliations:** 1 The UC Berkeley‐UCSF Graduate Program in Bioengineering, Berkeley, CA USA; 2 Department of Bioengineering, University of California, Berkeley, Berkeley, CA USA; 3 Innovative Genomics Institute, University of California, Berkeley, Berkeley, CA USA; 4 Department of Biochemistry and Biophysics, Center for Phage Technology, Texas A&M University, College Station, TX USA; 5 Environmental Genomics and Systems Biology Division, Lawrence Berkeley National Laboratory, Berkeley, CA USA; 6 Present Address: Armata Pharmaceuticals, Inc., Marina Del Rey, CA USA

**Keywords:** Bacteria, Mechanism of action, High-throughput screening, Genetics

## Abstract

Single-strand RNA (ssRNA) *Fiersviridae* phages cause host lysis with a product of single gene (*sgl* for single-gene lysis; product Sgl) that induces autolysis. Many different Sgls have been discovered, but the molecular targets of only a few have been identified. In this study, we used a high-throughput genetic screen to uncover genome-wide host suppressors of diverse Sgls. In addition to validating known molecular mechanisms, we discovered that the Sgl of PP7, an ssRNA phage of *Pseudomonas aeruginosa*, targets MurJ, the flippase responsible for lipid II export, previously shown to be the target of the Sgl of coliphage M. These two Sgls, which are unrelated and predicted to have opposite membrane topology, thus represent a case of convergent evolution. We extended the genetic screens to other uncharacterized Sgls and uncovered a common set of multicopy suppressors, suggesting that these Sgls act by the same or similar mechanism.

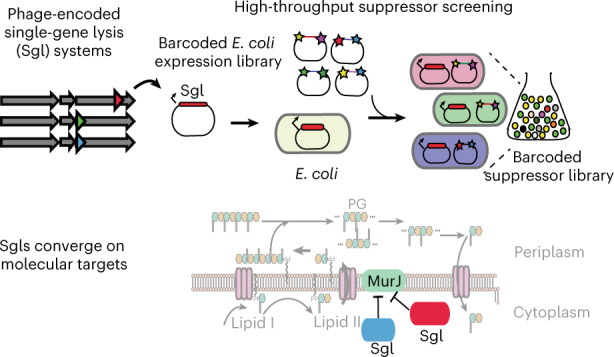

## Abstract (web summary)

In contrast to dsDNA phages where multiple genes are involved in programmed host lysis, ssRNA *Fiersviridae* phages require only a single gene. Here, genome-wide host suppressors of diverse single-gene lysis systems are identified using a high-throughput genetic screen.

## Main

Lysis of the bacterial host is the last step in the bacteriophage (phage) life cycle, determined by the lytic program encoded on the phage genome. In double-strand DNA (dsDNA) phages, lysis is mediated by multiprotein systems that disrupt the cytoplasmic/inner membrane, degrade the peptidoglycan (PG)/cell wall, compromise the outer membrane and regulate the lytic process^1^. In contrast, single-strand RNA (ssRNA) phages and lytic single-strand DNA (ssDNA) phages use the product of a single gene (*sgl*; product Sgl) to carry out host lysis^2,3^.

**Fig. 1:**
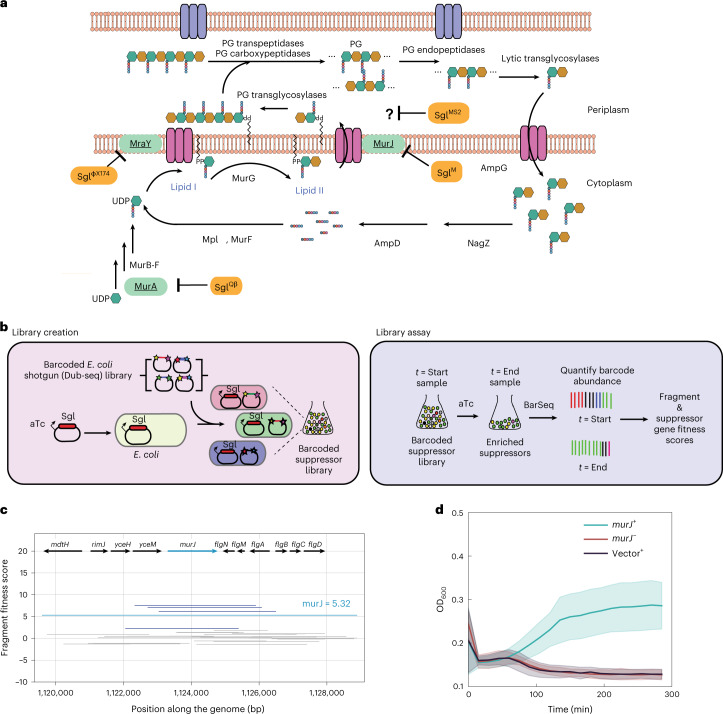
Genome-wide screen to identify host suppressors of phage-encoded single-gene lysis systems. **a**, Schematic representation of PG maturation and recycling in *E. coli*^29^. Three well-studied Sgl proteins (orange) are shown with their primary targets (green). The PG biosynthesis, translocation and regulation of PG maturation are reviewed in detail elsewhere^28^. **b**, Cartoon description of suppressor library creation and assay. A toxic gene (in this paper, an *sgl* gene) is cloned into an aTc-inducible vector. The Dub-seq library^15^ containing previously mapped dual-barcoded (shown as stars on the plasmid) random genomic fragments (shown as colored regions between stars) from BW25113 *E. coli* strain is transformed into the DH10B *E. coli* strain carrying the cloned *sgl* gene^15^, creating a barcoded suppressor library. Strains were tracked by quantifying the abundance of DNA barcodes associated with each strain by Illumina sequencing. Sgl-specific strain fitness profiles were calculated by taking the log_2_ fold change of barcode abundances between post- (*t* = End) and pre- (*t* = Start) induction of *sgl* and fragment and gene fitness scores calculated as described in Methods. **c**, Representative fragment and gene fitness data from our suppressor screening experiment for Sgl^M^. Dub-seq fragment (strain) data (*y* axis) for the genomic region (*x* axis) surrounding *murJ* under induction of Sgl^M^ is shown. Each purple and gray line is a Dub-seq fragment. Those that completely cover *murJ* are shown in purple, and fragments that do not contain *murJ* or cover partially are colored gray. The *murJ* gene fitness score of 5.32, estimated using a regression model, is shown as a blue line (Methods)^15^. Multiple barcodes representing fragments containing MurJ were specifically enriched in our Sgl^M^ screens. **d**, Growth curves show that heterologous expression of wild-type MurJ can suppress Sgl^M^ lytic activity. Teal represents *sgl*^*M*^ and *murJ* co-overexpression using aTc and IPTG, respectively. Red represents *sgl*^*M*^ expression in the absence of *murJ* induction using aTc without IPTG. Black represents *sgl*^*M*^ expression in the presence of an empty ASKA vector using aTc and IPTG. All curves are plotted as mean of three (*n* = 3) independent biological replicates surrounded by the 95% confidence intervals. Source data

Until recently, only 11 *sgl*s had been identified. Of these 11, the Sgls of the coliphages ΦX174, Qβ and M were shown to block the production and translocation of periplasmic lipid II, the universal precursor for PG synthesis, by inhibiting the conserved enzymes MraY, MurA and MurJ, respectively (Fig. 1a). The situation is mysterious, however, for the canonical male-specific coliphage MS2, which, in 1976, was the first genetic entity to have its complete genome published^4^. Mutational analysis revealed that a cryptic 75-codon reading frame was required for lysis^5^. This Sgl, named L, caused lysis when expressed alone from a plasmid vector; subsequent studies reported it to be a membrane protein^6^ and to support lysis without inhibiting net PG biosynthesis, as measured by incorporation of ^3^H-mDAP^7^. More recent genetic analysis has shown that L function requires formation of a complex with the chaperone DnaJ^8^. Moreover, mutational analysis and comparison with other Sgls led to the hypothesis that the other seven known Sgls were ‘L-like’, in that, despite the lack of sequence similarity, they shared a characteristic three-domain structure, including a characteristic Leu-Ser motif at the C-terminus of a hydrophobic domain^9^. On this basis, it was proposed that the L-like Sgl family shared a common target, conserved in their diverse bacterial hosts (*Pseudomonas*, *Acinetobacter*, *Caulobacter* and *Escherichia coli*).

Interest in the Sgl field suddenly increased when, beginning in 2016, environmental metagenome and transcriptome mining increased the available sequence diversity of *Fiersviridae* to upwards of 10,000 genomes^10–12^. Although the hosts of these ssRNA phages are unknown, each genome is expected to encode at least one Sgl. The prospect of identifying thousands of diverse Sgls, each with the capability of finding a ‘weak spot’ in bacterial cell wall biogenesis, is alluring, not the least because it might lead to opportunities for antibiotic development. However, the classic molecular genetic approach has required decades to find three Sgl targets and has left the L mechanism still enigmatic. Thus, scalable forward genetic screening platforms may be required to realize the promise and diversity of Sgls^13,14^. One approach to this problem may be Dub-seq, which is founded on doubly barcoded overexpression libraries of randomly sheared bacterial DNA^15^. Once the barcodes are mapped to chromosomal segments, the Dub-seq library can be used for competitive fitness assays^15^. We have demonstrated the utility, scalability and barcode standardization of gain-of-function fitness assays by studying the tolerance phenotypes against diverse antibiotics, stressors and metals and, most recently, characterized genetic barriers in phage–host interactions in *E. coli*^15,16^.

In this study, we repurposed Dub-seq for genome-wide assessment of host suppressors of Sgl activity and applied it to five diverse ssRNA phage Sgls awaiting molecular target characterization. We established the screening platform by recapitulating the known molecular target of Sgl of coliphage M. The results enable a rapid determination of one of the Sgl targets and suggest common or similar mechanisms for most of the others.

## Results

### Devising rapid Sgl suppressor identification screens

To accommodate the recent expansion of the Sgls^14^, here we use a systematic nomenclature format consistent with previous work^3^. For instance, the Sgl from phage M is represented as Sgl^M^, and Sgl L from MS2 is Sgl^MS2^.

Previously, Sgl targets have been identified by inducing a multicopy plasmid clone of the *sgl* and selecting for spontaneous missense mutations in the target genes^17–19^ or for multicopy suppressors using an *E. coli* gene library (as in the case of Sgl^M^)^19^. Both approaches are constrained, the former for availability of mutable sites that block Sgl/target interaction without destroying function and the latter for appropriate Sgl/target copy number and affinities. We hypothesized that expressing an *sgl* gene in the context of a barcoded shotgun expression library of the host (for example, *E. coli* Dub-seq library expressed in *E. coli*) and using barcode sequencing as a readout would enable the quick identification of all genes in a genome that contribute to fitness, including the target gene, even if Sgl/target levels were not ideal (Fig. 1b).

As a proof of principle, we adapted our Dub-seq platform for screening suppressors against the toxicity of Sgl^M^, an inhibitor of the lipid II flippase MurJ (Fig. 1a–d)^19^. Sgl^M^ was chosen because the first evidence for its targeting MurJ came from a multicopy suppression of induced *sgl*^*M*^ lysis using an *E. coli* gene library^19^. In brief, we cloned *sgl*^*M*^ into a low-copy plasmid under an anhydrotetracycline (aTc)-inducible promoter and showed that induction caused lysis in *E. coli* K-12 (Fig. 1d and Methods). We then moved a previously characterized *E. coli* Dub-seq plasmid library (pFAB5516), consisting of *E. coli* genomic DNA fragments cloned between two 20-bp random DNA barcodes into our assay strain DH10B *E. coli* cells^15^. This process resulted in a library of 17,007 unique members (BA1320L) with each strain harboring an inducible *sgl*^*M*^ vector and a unique member of the pFAB5516 library (Fig. 1b and Supplementary Table 1). We then subjected this BA1320L library to Sgl^M^ induction in liquid culture, isolated plasmid DNA from cells collected before and after induction and subjected the DNA to BarSeq polymerase chain reaction (PCR). The product was sequenced on a HiSeq 4000 platform (Methods) and analyzed for the change in barcode abundance (a proxy for cloned genomic region consisting of ~1–3 genes) after Sgl^M^ induction. We then calculated the fragment fitness score for each strain by taking the normalized log_2_ ratio of the number of reads for each barcode at the end and at the start of the experiment (Fig. 1b). Positive fragment scores indicate that the gene(s) contained on that fragment lead to an increase in relative fitness, whereas negative scores mean the gene(s) on the fragment cause reduced relative fitness. To account for causative and non-causative genes on each fragment, we use a regression model to examine the score of all fragments containing the gene and compute gene fitness scores (Methods)^15^. We classified genes with a fitness score >3 as high-confidence hits if they have sufficient read coverage (>25 reads per barcode for both *t* = 0 and the experiment), and these fitness effects were consistent across multiple fragments that cover the genes and across replicate experiments (Methods). We observed that induction of *sgl*^*M*^ yielded reproducible data (*n* = 2 fitness experiments; Supplementary Fig. 1). As expected, *murJ* emerged as a consistently enriched gene covered by multiple fragments in our screen (Fig. 1c and Supplementary Fig. 1); moreover, heterologous expression of wild-type *murJ* suppressed the lytic activity of induced *sgl*^*M*^ (Fig. 1d). These experiments indicated that the Sgl-dependent growth defect coupled with Dub-seq suppressor screen could correctly identify host factors that, when overexpressed, overcome the toxicity of the Sgl protein and, thus, could map the Sgl target to host pathways.

### Extending Dub-seq suppressor screens to additional Sgl genes

**Fig. 2:**
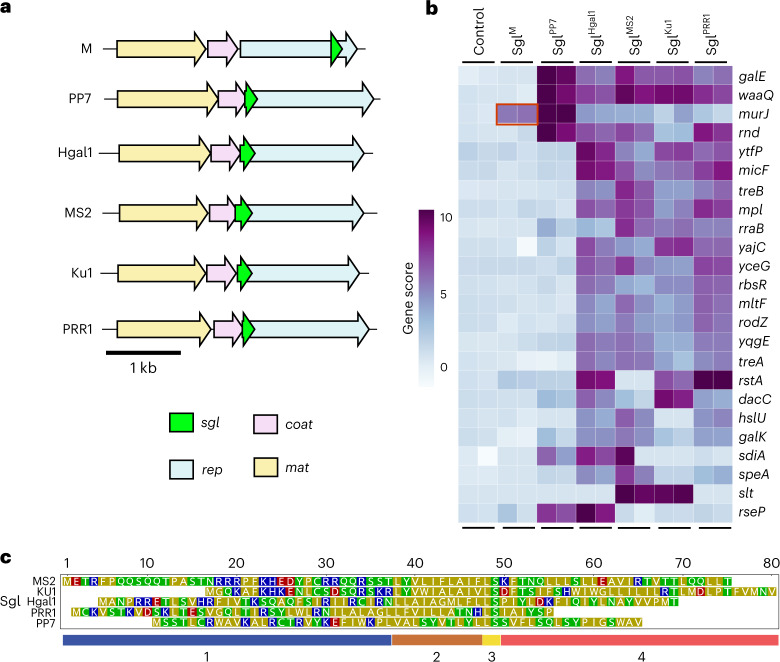
Sgl diversity in genomic context, sequence identity and suppressor genotypes. **a**, All *Fiersviridae*-derived Sgls investigated in this study are shown within their native genomic context as a schematic. All lysis genes (*sgl*, green) occur in sequences overlapping with one or more additional genes; *mat* encodes the maturation protein responsible for adsorption to the receptor pilus, *coat* encodes the capsid protein and *rep* encodes the replicase. **b**, Multicopy suppressors of lysis proteins as identified through high-throughput gain-of-function screening. A selection of high-confidence, top-scoring genes is shown for visualization purposes from two biological replicate (*n* = 2) independent library experiments performed per *sgl* suppressor experiment. Multicopy suppressor identified from previous work is boxed^19^ in red. **c**, Sequence alignment of *Fiersviridae* lysis proteins investigated here shows that they bear little resemblance to each other. Sequence alignment was done manually^9^. Acidic and basic residues are in red and blue, respectively, whereas polar and non-polar residues are shown in green and yellow, respectively. Domains proposed for L-like Sgls previously^9^ are outlined at the bottom of the diagram. In brief, domain 1 (blue) refers to the variable-length, positively charged N-terminus; domain 2 (brown) refers to the LS-preceding hydrophobic region; domain 3 (yellow) refers to the L-like conserved LS motif; and domain 4 (red) refers to the highly variable C-terminus. Source data

The success of using Dub-seq to identify MurJ as the target of Sgl^M^ encouraged us to apply the method to Sgl^MS2^, the L protein, which has remained mechanistically uncharacterized for nearly half a century. Moreover, we included in the test set the Sgls from four other ssRNA phages: KU1, Hgal1, PRR1 and PP7 (Fig. 2a). This set represents a spectrum of diversity in terms of the cellular environment within which the Sgl must function and, in aggregate, represents six different genera within the *Fiersviridae* family (Supplementary Table 2). KU1 is F-specific and, thus, restricted to *E. coli* and closely related *Enterobacteriaceae*. Both Hgal1 and PRR1 use the conjugative pili of multidrug resistance plasmids as receptors and, thus, must function in rather diverse host environments. In contrast, PP7 recognizes the polar pilus of *Pseudomonas*^2^. Overall, our test set Sgls are from phages that are specific for a particular retractable pilus, the genes for which are often located on mobile genetic elements^20^. Therefore, it is highly likely that an ssRNA phage may require the ability to propagate in a broad range of host cytoplasms. Thus, it is not unreasonable to expect that at least some functionality is preserved in heterologous hosts. In any case, as noted above, all five Sgls in this test set are proposed to be ‘L-like’ Sgls and, thus, expected to have the same cellular target, based on sharing the three domains plus an apparent LS motif organization revealed in the mutational analysis of MS2 lysis protein L^9^.

In total, we performed 12 genome-wide suppressor screens for these five Sgls (and a control), collected suppressor candidates and processed BarSeq PCR samples for deep sequencing (Methods; experimental and library overviews are described in Supplementary Data 1 and 2, respectively). After curation of the dataset for sufficient read coverage and consistency, we identified 190 high-confidence hits encompassing 96 genes across the five suppressor Dub-seq screens (Fig. 2b and Methods; complete library read counts, fragment scores and gene scores are presented in Supplementary Data 3–5, respectively). Thus, about 2% of genes exhibited at least one suppression phenotype.

More striking is the number of suppressor genes identified (96 total) for a group of four Sgls: MS2 (38 genes), KU1 (36 genes), Hgal1 (55 genes) and PRR1 Sgls (55 genes). Forty-five genes suppress at least two of these four Sgls, of which 22 suppress at least three, and ten suppress all four (Supplementary Data 5 and Supplementary Figs. 2–6). In contrast, Sgl^PP7^ toxicity was suppressed by only six genes. Two genes, *waaQ* and *galE*, appeared as multicopy suppressors of all five Sgls. We assert that neither *galE* nor *waaQ* is likely to be the target of any of these Sgls and, instead, are genes that, when overexpressed, indirectly mitigate Sgl toxicity. The assay strain that we used in this work is a *galE* mutant, and overexpression of wild-type *galE* probably provides fitness benefits to the cell under Sgl-induced toxicity. Furthermore, overproduction of GalE, which codes for UDP-glucose-4-epimerase, has been shown to provide fitness benefits in earlier genetic screens, probably playing a role in modulating outer membrane (OM) biogenesis^15,21^. The fragments carrying *waaQ* would also produce the RirA small RNA (sRNA), which activates the transcription of *rpoE* encoding the sigma factor for genes involved in periplasmic and OM maintenance^22^. Neither *waaQ* nor *rpoE* knockouts have overt lytic phenotypes but have been shown to exhibit some sensitivity to detergents and a plant-based antibacterial agent^23^.

**Fig. 3:**
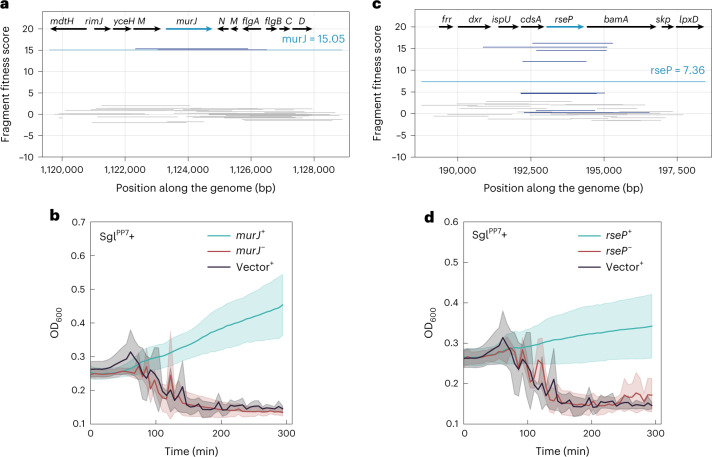
Sampling of validated multicopy suppressors against Sgl protein expression. **a**,**b**, Panels refer to suppressor activity of *murJ* expression against Sgl^PP7^-mediated lysis. **c**,**d**, Panels refer to suppressor activity of *rseP* expression against Sgl^PP7^-mediated lysis. Dub-seq fragment plots for the highlighted suppressor locus are shown in **a** and **c**. Dark blue lines correspond to fragments covering the gene of interest. Gray lines correspond to fragments not covering the gene of interest. Teal line corresponds to gene fitness score (Methods). **b**,**d**, Panels show lysis inhibition growth effects from a 96-well microplate reader assay. Multicopy suppressors or empty vector controls were expressed from the corresponding ASKA mutant collection plasmid under IPTG control. Teal curves correspond to suppressor induction at 50 µM IPTG. Black curves correspond to the empty ASKA vector negative control. Red curves correspond to the uninduced suppressor plasmid. All curves are plotted as mean of three (*n* = 3) independent biological replicates surrounded by the 95% confidence intervals. Large variations in optical density are caused by the aggregation of viscous cell debris observed in the course of the microplate reader experiment. Source data

Although Sgl^PP7^ and Sgl^M^ shared no substantial sequence similarity with each other (Fig. 2c and Supplementary Fig. 7; 15.6% sequence identity, MUSCLE BLOSUM62 matrix^24^), suppressor assays with both Sgls yielded high-scoring *murJ*-containing fragments (Figs. 1b and 3a). We wondered if MurJ could also be the target of Sgl^PP7^. To validate the MurJ suppression of Sgl^PP7^, we transferred the *murJ*-expressing plasmids from the ASKA collection into *E. coli* and tested for the ability to inhibit Sgl^PP7^ lysis in liquid culture after induction with aTc (for the *sgl*) and isopropyl β-d-1-thiogalactopyranoside (IPTG) (for the candidate target gene) (Fig. 3b and Methods)^25^. The results clearly show that the multicopy *murJ* clone from the ASKA plasmid library can block lysis in an induction-specific fashion; that is, it is not just the presence of the multicopy gene that affects suppression. A similar lysis inhibition result was obtained for another hit, *rseP* (Fig. 3c,d), which encodes a protease known to cleave membrane proteins^26^. These results suggest that MurJ blocks lysis by titrating out the Sgl, whereas RseP acts to degrade it.

**Fig. 4:**
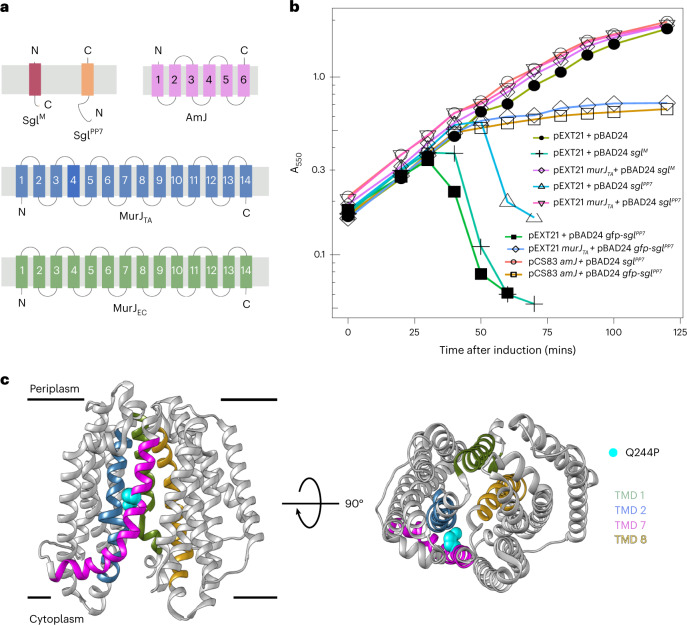
Sgl^PP7^–MurJ interaction. **a**, Predicted membrane topologies of Sgl^M^ (red), Sgl^PP7^ (orange), AmJ (*B. subtilis*) (pink), MurJ_TA_ (*T. africanus*) (blue) and MurJ_EC_ (*E. coli*) (green) are shown in the context of bacterial cytoplasmic membrane (gray rectangle) with periplasmic side and cytoplasmic side represented above and below the gray rectangle, respectively. The N-termini and C- termini of the respective proteins are indicated with ‘N’ or ‘C’. **b**, Lysis profiles of assay strain TB28 co-transformed with plasmids carrying inducible *sgl* genes (*sgl*^*M*^, *sgl*^*PP7*^ and *GFP-sgl*^*PP7*^) and compatible plasmids expressing MurJ orthologs (MurJ_TA_^35^ and AmJ^36^). These include pEXT21 + pBAD24 (emplty vector control, dark olive and filled circle), pEXT21 + pBAD24 *sgl*^*M*^ (topaz and cross), pEXT21 *murJ*_*TA*_ + pBAD24 *sgl*^*M*^ (light purple and diamond), pEXT21 + pBAD24 *sgl*^*PP7*^ (cerulean and triangle), pEXT21 *murJ*_*TA*_ + pBAD24 *sgl*^*PP7*^ (hot pink and inverted triangle), pEXT21 + pBAD24 *gfp-sgl*^*PP7*^ (dark mint green and filled square), pEXT21 *murJ*_*TA*_ + pBAD24 *gfp-sgl*^*PP7*^ (soft blue and diamond), pCS83 *amJ* + BAD24 *sgl*^*PP7*^ (salmon and open circle) and pCS83 *amJ* + pBAD24 *gfp-sgl*^*PP7*^ (ochre and open square). **c**, Representative lysis profiles from three (*n* = 3) independent biological replicates are shown. The amino acid substitutions in *E. coli murJ* (MurJ_EC_) that confer resistance to *gfp–sgl*^*PP7*^ are shown on the crystal structure of an inward open conformation of MurJ_EC_ (Protein Data Bank: 6CC4). The TMDs that line the central hydrophilic cavity are colored as follows: TMD1 (olive drab), TMD2 (steel blue), TMD7 (magenta) and TMD8 (gold rod). The substituted amino acid is highlighted as cyan spheres on TMD7 (magenta). Lateral view (left) and periplasmic view (right). Source data

To further investigate the activity and specificity of MurJ suppression of Sgl^PP7^-induced lysis, we co-expressed Sgl^PP7^ with the lipid II flippases MurJ_TA_ from *Thermsipho africanus* or Amj from *Bacillus subtilis* (Fig. 4a,b). The results indicate that Sgl^PP7^ lethality can be rescued by expression of heterologous lipid II flippases, which strongly suggests that Sgl^PP7^ targets MurJ. Moreover, we obtained unambiguous evidence for the Sgl^PP7^–MurJ interaction using a genetic approach. We constructed a fusion gene, *gfp–sgl*^*PP7*^, that exhibited enhanced lytic function (Fig. 4b), allowing us to select spontaneous mutants that survived the induction of the fusion gene (Fig. 4b). Analysis of the survivors revealed a single amino acid substitution in MurJ conferring Sgl^PP7^ resistance: Q244P (Fig. 4c). This missense change is localized to transmembrane domain 7 (TMD7), one of the 14 transmembrane domains that define the solvent-exposed cavity of MurJ and, specifically, undergo a major conformation shift as MurJ alternates between cytoplasmic-open and periplasmic-open states^19,27^. Considering that this amino acid change was previously observed to confer resistance to Sgl^M^, these two dissimilar proteins may target the same molecular interface of MurJ^19^.

### Suppressor patterns for the L-like Sgl proteins

In contrast to Sgl^M^ and Sgl^PP7^, where MurJ is one of the few genes identified as a suppressor, the suppressor profiles for the presumptive L-like Sgls from MS2, KU1, Hgal1 and PRR1 are complex (Fig. 2b, Supplementary Figs. 2–6 and Supplementary Data 5). Aside from *waaQ* and *galE*, ten genes showed high fitness scores, indicating that they play a role in mitigating the toxicity induced by Sgls: *micF*, *mltF*, *mpl*, *rodZ*, *rbsR*, *treB*, *yajC*, *yceG*, *yqgE* and *ytfP*. *mltF* and *yceG* encode lytic transglycosylases involved in PG turnover^28–30^. Mpl is a murein peptide ligase involved in recycling the PG precursors derived from cell wall turnover^31,32^. By providing an alternate source of PG precursors, these three could provide palliative relief indirectly to any developing insult to cell wall biosynthesis and turnover (Fig. 1a). MicF is an sRNA and has been shown to regulate OM porin expression and can, thus, influence the integrity and permeability of the envelope^22^. Other hits are more difficult to rationalize (Discussion). YajC is part of the Sec translocon accessory complex but of unknown function. RbsR is a repressor of ribose catabolism and transport. RodZ is a key regulator of cell division, interacting directly with the FtsZ septal ring. TreB is part of the phosphotransferase system pathway for trehalose import. None of these high-scoring genes is essential^33^, and in none of these cases is there a lytic phenotype associated with a gene knockout. The simplest idea is that all of these suppressor fragments exert indirect effects or act through sRNAs such as MicF and RirA (from processing of the waaQ transcript)^22^, many of which likely remain cryptic.

In addition to the broadly high-scoring genes mentioned above, we also uncovered high fitness scores for a few genes that are specific for some L-like Sgls (such as *hslVU*, *rstA*, *dacC*, *slt*, *sdiA*, *speA* and *galK*; Fig. 2b), and the specific pattern of these scores is difficult to rationalize. For example, high scores of *slt* (encoding the main lytic murein transglycosylase) and *dacC* (codes for d-alanyl-d-alanine carboxypeptidase) makes sense as they are considered to be playing a role in PG quality control pathways, but the specific fitness effect of *slt* in the KU1 and MS2 assays compared to that of *dacC* for the Sgls of KU1, Hgal1 and PRR1 dataset is intriguing. It should be noted that the genes encoding a protease active against membrane proteins (for example, HslVU) are overrepresented in the suppressor collection for three out of five L-like Sgls but not for Sgl^M^ or Sgl^PP7^ (MS2, Hgal1 and PRR1; Fig. 2b). This may indicate that L-like Sgls largely remain sensitive to proteases during the lytic pathway, possibly because they do not form a stable complex with a protein target. Overall, the conclusion is that the lytic function of these four L-like Sgls can be suppressed by multicopies of many genes involved in envelope homeostasis. Finally, although DnaJ has been shown to interact with the N-terminal (domain 1) of MS2 L^8^, deletion of this domain eliminates DnaJ dependency for L function, strongly suggesting that the DnaJ–L interaction is a regulatory feature rather than part of the core lytic pathway. Moreover, domain 1 of the other L-like Sgls is not conserved (Fig. 2c), and, therefore, they may not require DnaJ. Nevertheless, it is possible that other Sgls, L-like or not, may also be dependent on protein chaperones other than DnaJ and that this would complicate identification of the target protein.

## Discussion

In this study, we applied a genome-wide genetic screen to identify multicopy suppressors of Sgl lysis proteins—encoded by a diverse group of lysis genes (*sgls*) from phages belonging to the same (*Fiersviridae*) family but different genera (Supplementary Table 2). As a proof of principle, we benchmarked our genetic screen against the toxicity of Sgl^M^, and we recapitulated the identification of its known target, the lipid II flippase MurJ, as the high-confidence candidate (Fig. 1c,d)^19^. Encouraged with this result, we applied the method to Sgl^MS2^, the L protein, whose target has remained enigmatic over half a century. In addition to Sgl^MS2^, we also screened Sgls from four other ssRNA phages—KU1, Hgal1, PRR1 and PP7—that share the characteristic four-motif structure of L protein and, therefore, were previously proposed to have the same cellular target. In total, we observed 190 high-confidence hits across the *sgl*^*M*^, *sgl*^*PP7*^, *sgl*^*PRR1*^, *sgl*^*MS2*^ and *sgl*^*Hgal1*^ Dub-seq screens. We followed up with one of the top-scoring candidates for Sgl^PP7^ and confirmed that Sgl^PP7^ lethality can be rescued by expression of heterologous lipid II flippase MurJ, strongly suggesting it to be a target of Sgl^PP7^. Thus, it appears that, functionally, Sgl^PP7^ is not an L-like Sgl. Overall, the Dub-seq genetic screen successfully uncovered high-confidence multicopy suppressors that play a role in PG biosynthesis or are known to alleviate OM stress response and provide insights into Sgl target/repair pathways at a genome-wide scale that may be challenging to obtain via traditional approaches.

One of the major unexpected findings of this study was that the Sgl^PP7^ targets MurJ, an essential lipid II flippase in Gram-negative bacteria. Interestingly, the Sgl^PP7^ has no primary structure resemblance or genomic synteny to the other MurJ-targeting Sgl, Sgl^M^. Furthermore, the lytic function of these two Sgls was blocked by the same single missense change (Q244P) in MurJ, suggesting that these two disparate Sgls have not only convergently evolved to target the same protein but may also target the same molecular interface on MurJ. The resistance allele Q244P is located on TMD7, one of the four TMDs lining the central hydrophilic cavity of MurJ. Interestingly, TMD7 undergoes large conformational changes between periplasmic-open and cytoplasmic-open states of MurJ, and Gln244 is positioned at the bend in the helix. Locking MurJ in either of the conformational states leads to accumulation of lipid II in the inner leaflet of the inner membrane and ultimately results in cell lysis. Previously, cysteine accessibility studies (SCAM) have shown that Sgl^M^ locks MurJ in periplasmic-open conformation and blocks the transfer of lipid II across the membrane. Given the putative interaction interface of Sgl^PP7^ at the highly dynamic TMD7 of MurJ, potentially Sgl^PP7^ locks MurJ in the opposite conformation to Sgl^M^—that is, cytoplasmic-open conformation. Future SCAM analysis and structural studies of Sgl^PP7^–MurJ complex should shed light on both the conformation state of MurJ–Sgl^PP7^ complex and its interaction interface.

As noted earlier, we acknowledge that many of the candidate suppressors uncovered by our screen on Sgls are difficult to rationalize mechanistically. Although we have not tested all suppressor hits as individual candidates, the suppressor list generated here should be regarded as a starting point for future mechanistic studies. A few caveats are worth noting. Changes in growth rate, metabolism, envelope biosynthesis and stress response that indirectly suppress Sgl lytic function may arise due to high gene dosage. Finally, it is possible that some of these hits are not biologically relevant in vivo—that is, the products of those genes might not suppress the Sgl phenotype in the context of phage infection. The most obvious reason for this would be a mismatch between expression levels of the Sgl gene in our plasmid vector and in the context of the infected cells. Both physiological and gene expression differences of Sgl could mediate the impact of our found suppressors. Nonetheless, the fact that we found known validated suppressors along with a list of genes with similarly strong suppressor effects of our Sgls is compelling.

Recently, the number of experimentally validated Sgls has expanded by 35, and they share no detectable similarity to the previously characterized Sgls^14^. The high sequence diversity of Sgls naturally implies possible diversity in molecular targets to affect host cell lysis. However, we speculate that evolution of Sgls is highly constrained as being part of compact ssRNA phage genomes, in addition to being commonly found as overlapped or encoded within other phage genes. Also, being membrane associated probably makes them target other membrane proteins, such as PG proteins localized in the inner membrane. Hence, convergent evolution of Sgls to target the limited number of host targets is an inevitable consequence, and one should expect more cases of convergent evolution to the known targets, such as MurA, MraY and MurJ. We note here that the recently reported 35 Sgls were selected for having a predicted TMD domain, so it is possible that they are likely biased toward interacting with MurJ and MraY. The fact that both Sgl^M^ and Sgl^PP7^ target MurJ suggests that there is more than one way to exploit the same ‘weak spot’ in the bacterial cell wall machinery. Furthermore, a target uncovered in one species of bacteria (that is, MurJ in *Pseudomonas*) could also serve as one in another more distant species (that is, MurJ in *E. coli*). Thus, by studying convergently evolved Sgls, one could gain insights into built-in universal molecular ‘weak spots’ across various species.

We limited this study to the discovery of suppressors for unique Sgl lysis proteins of *Fiersviridae*. We anticipate this forward genetic screening approach to be generalizable and extendable to discover suppressors of many other toxic genes found in nature, including Sgls from the recent hyperexpansion of ssRNA phage genomes. Furthermore, this approach could be useful in the study and annotation of dsDNA and ssDNA phage genomes and host-encoded small toxic genes^34^. We demonstrate here that repurposing Dub-seq technology for carrying out high-throughput suppressor screens will greatly expedite hypothesis generation and target identification of Sgl lysis proteins, providing a new avenue for antibiotic and phage-derived biotechnological discovery.

## Methods

### Bacterial strains and growth conditions

In general, all *E. coli* strains were grown at 37 °C and 180 r.p.m. in Lysogeny Broth (LB-Lennox broth, Sigma-Aldrich) supplemented with antibiotics, unless stated otherwise. When appropriate, 50 µg ml^−1^ kanamycin and/or 34 µg ml^−1^ chloramphenicol (denoted with +K or +C, respectively) were added to media. All bacterial strains and libraries were stored at −80 °C for long-term storage in 25% sterile glycerol (Sigma-Aldrich). All library assays were performed in NEB 10-beta strain backgrounds (*araD*139 Δ(*ara-leu*)7697 *fhuA lacX74 galK* (Φ80 Δ*(lacZ)M15) mcrA galU recA1 endA1 nupG rpsL* Δ*(mrr-hsdRMS-mcrBC)*, New England Biolabs). A complete list of strains and plasmids is provided in Supplementary Data 6. A list of primers and gene sequences used in this work is provided in Supplementary Data 7.

### Construction of *sgl* expression strains

Template sequences for *sgl*^*Hgal1*^, *sgl*^*M*^, *sgl*^*MS2*^, *sgl*^*PRR1*^ and *sgl*^*PP7*^were identified from the NCBI-deposited genomes: NC_019922, NC_019707, NC_001417, NC_008294 and NC_001628, respectively. As a toxic gene control, we used protein PC02664 detected from a phage genome infecting *E. coli*^37,38^. Each gene was codon-optimized for *E. coli*, had BsaI sites removed and was synthesized de novo (Integrated DNA Technologies (IDT), GenScript and Twist Bioscience). *Sgl* genes were cloned into pBA368, a Golden Gate *gfp* dropout vector originally derived from pBbA2K-rfp (Addgene plasmid 35327). DNA assembly was performed via Golden Gate assembly using BsaI (New England Biolabs), pBA368 and one of the synthesized *sgl*s. Reactions were cleaned up using DNA Clean and Concentrate (Zymo Research), transformed into NEB 10-beta competent cells (New England Biolabs) and plated on LB + K. GFP^−^ colonies were picked, grown up, stored at −80 °C and verified for the intact *sgl*.

For all strains, lytic activity was measured via plate reader assay before constructing suppressor libraries. Strains were inoculated into LB + K media overnight. Cells were diluted 50× into LB + K media with varying levels of aTc ranging from 0 ng ml^−1^ to 200 ng ml^−1^ in a flat-bottom 96-well plate (Corning, 3904). Sgl-mediated lysis progressed in Tecan Infinite F200 readers with orbital shaking and OD_600_ readings every 15 minutes for 3–5 hours at 37 °C. Strains with functional Sgl phenotypes typically had visible lysis after ~90 minutes.

Plasmid pBAD24-*sgl*^*PP7*^*-lacZ*_*α*_ was constructed in multiple steps. First, the *sgl*^*PP7*^ (NC_001628.1) was codon-optimized for *E. coli* expression (IDT), and a synthetic DNA construct was obtained (GenScript). The synthetic *sgl*^*PP7*^ DNA was amplified using primers KC94 and KC116, and the resulting PCR product was gel-purified (Qiagen), digested with restriction enzymes EcoRI and XhoI (New England Biolabs) and subcloned into plasmid pKC3, replacing *sgl*^*M*^ in pKC3.

Plasmid pBAD24-*gfp-sgl*^*PP7*^*-lacZ*_*α*_ was constructed via the overlap extension PCR method using primers KC36 and KC127 to amplify a *gfp* megaprimer in the first PCR^39^. The megaprimer was then used to insert *gfp* into pBAD24-*sgl*^*PP7*^*-lacZ*_*α*_ plasmid during the second PCR. The product of the second PCR reaction was treated with DpnI and then transformed into competent XL1Blue cells. The constructs were verified by sequencing (Eton Biosciences) with primers KC30 and KC31.

### Construction of Dub-seq suppressor libraries

Here, we sourced previously constructed *E. coli* (BW25113) Dub-seq library (pFAB5516)^15^ for building Dub-seq suppressor libraries. As reported earlier, the average fragment size in *E. coli* (BW25113) Dub-seq library is 2.6 kb with 2–3 genes covered completely. More than 95% of all genes are covered in the library by at least one fragment, and just 135 genes are not covered in their entirety. To build Dub-seq suppressor libraries, we transformed the plasmid Dub-seq pFAB5516 library^15^ directly into *sgl* expression strains (above) via electroporation. Competent cells were created from an overnight culture diluted 70× into 25 ml of LB + K, grown at 37 °C and 180 r.p.m. for ~3 hours until OD_600_ was 0.5–0.7. The resulting mid-log cultures were chilled at 4 °C. Cultures were centrifuged (Beckman Coulter, Allegra 25R) for 5 minutes at 8,000*g* and subjected to three washes: (1) once with 25 ml of chilled water and (2) twice with 15 ml of chilled 10% glycerol. The cell pellets after the final glycerol wash were resuspended in 10% glycerol, yielding ∼250 µl of cells.

For each *sgl* library, five parallel transformations were performed to minimize inefficiency bias from any individual transformation. Each transformation consisted of 40 µl of competent cells and 10 ng of the pFAB5516 plasmid library transferred to a chilled cuvette (1-mm gap, VWR). Cuvettes were electroporated using a BTX-Harvard Apparatus ECM 630 Exponential Decay Wave Electroporator with the following parameters: voltage (1,800 V), resistance (200 Ω) and capacitance (25 μF). After each transformation, cells were recovered in 1 ml of LB + K media at 37 °C for 1 hour. For each transformation, 980 µl of each recovery was plated and spread out onto LB + K + C agar in a 245-mm × 245-mm bioassay dish (Nunc). The remaining 20 µl of cells was serially diluted and plated onto a standard LB + K + C agar plate to estimate the number of transformants per electroporation. All transformations were incubated at 37 °C overnight.

After overnight incubation at 37 °C, we first quantified the transformations to ensure that we had at least 250,000 total estimated colonies (that is, ≥5× pFAB5516 library coverage). We then picked ten colonies from each of the transformations, carried out PCR and followed by Sanger sequencing to ensure that the *sgl* was free of mutations. If any *sgl* mutations were detected in this subset, we repeated the library construction. The transformant colonies were scraped and resuspended in 25 ml of LB + K + C media and processed as described above to make multiple 1-ml −80 °C freezer stocks^15^. Because pFAB5516 was characterized earlier^15^, there was no need to perform library mapping PCRs at this step. An overview of the suppressor library composition is summarized in Supplementary Table 1, and a gene-level description is shown in Supplementary Data 2.

### Liquid culture fitness experiments

Competitive fitness experiments were performed in liquid culture with two replicate experiments performed per *sgl* suppressor experiment. In brief, a 1-ml aliquot of suppressor Dub-seq library was gently thawed and used to inoculate 25 ml of LB + K + C media. The library culture was grown to an OD_600_ of ~1.0 at 37 °C. From this culture, two 1-ml pellets were collected, comprising the ‘Time 0’ or reference samples in BarSeq analysis. The remaining cells were diluted to a starting OD_600_ of 0.02 in LB + K + C media. A 690-µl volume of cells was mixed with 10 µl of diluted aTc (Sigma-Aldrich) and transferred to a 48-well microplate (700 µl per well) (Greiner Bio-One, 677102) and covered with breathable film (Breathe-Easy). For all experiments, unless otherwise noted, aTc was used at 15.6 ng ml^−1^. The progress of Sgl lysis was followed in Tecan Infinite F200 readers with orbital shaking and OD_600_ readings every 15 minutes for 8–12 hours at 37 °C. At the end of the experiment, the contents of each well were collected and spun down on a tabletop centrifuge to collect as a pellet individually. All pellets were stored at −80 °C until prepared for BarSeq (detailed below). A summary of all library experiments is described in Supplementary Data 1.

### BarSeq of Dub-seq pooled fitness assay samples

Plasmid DNA was isolated from stored pellets of enriched and ‘Time 0’ (‘time=zero’) Dub-seq samples using the QIAprep Spin Miniprep Kit (Qiagen). We performed 98 °C BarSeq PCR protocol as described previously^40^. BarSeq PCR in a 50-µl total volume consisted of 20 µmol of each primer and 150–200 ng of plasmid DNA. For the HiSeq 4000 runs, we used an equimolar mixture of four common P1 oligos for BarSeq, with variable lengths of random bases at the start of the sequencing reactions (2–5 nucleotides). Equal volumes (5 µl) of the individual BarSeq PCRs were pooled, and 50 µl of the pooled PCR product was purified with the DNA Clean and Concentrator Kit (Zymo Research). The final BarSeq library was eluted in 40 µl of water. The BarSeq samples were sequenced on Illumina HiSeq 4000 with 50 SE runs.

### Data processing and analysis of BarSeq reads

Fitness data for Dub-seq suppressor libraries were analyzed as previously described with a few modifications, using ‘barseq’ script from the Dub-seq Python library with default settings^15^. From a reference list of barcodes mapped to the genomic regions (BPSeq and BAGseq), and the barcode counts in each sample (BarSeq), we estimated fitness values for each genomic fragment using the ‘gscore’ script from the Dub-seq Python library. At this step, instead of pooling all Time 0 samples together, the Time 0 samples within each suppressor library were pooled, because the composition and abundance of library members between libraries was distinct. For instance, MS2 experiments had Time 0 samples different from those of the PP7 experiments. The ‘gscore’ script identifies a subset of barcodes mapped to the genomic regions that are well represented in the Time 0 samples for a given experiment set. A barcode was required to have at least ten reads in at least one Time 0 (sample before the experiment) sample to be considered a valid barcode for a given experiment set. The ‘gscore’ script was used to calculate a fitness score (normalized ratio of counts between the treatment sample and sum of counts across all Time 0 samples) for the strains with valid barcodes. From the fitness scores calculated for all Dub-seq fragments, a fitness score for each individual gene that is covered by at least one fragment was calculated using non-negative least squares regression as described previously^15^. The non-negative regression determines if the high fitness of the fragments covering the gene is due to that particular gene or its nearby neighboring gene and avoids overfitting. Raw data for reads, f-scores and g-scores across all experiments are provided in Supplementary Data 3–5, respectively.

We applied additional filters to ensure that the fragments covering the gene had a genuine benefit. In brief, we identified a subset of the effects to be reliable if the fitness effect was large relative to the variation between start samples (|score| ≥ 2) for both mean and gene fitness scores^15^; if the g-scores and f-scores appeared to be reproducible across replicate experiments; and if the number of reads for those fragments was consistently sufficient for the gene score to have little noise. Due to the strong selection pressure and subsequent fitness distribution skew resulting from Sgl activity, all candidate genes passing these filters were then subjected to manual scrutiny. For each gene, all barcodes were analyzed by f-score and reads. Several genes covered by few fragments (that is, ≤3) had inconsistent f-scores, with orders of magnitude different read depth. This bias yielded inflated g-scores and were discarded from further analysis. However, genes covered by individual fragments were kept in such cases.

### ASKA-based validations

To validate select lysis suppressor phenotypes from suppressor screens, we performed plate reader assays using additional plasmids derived from the overexpression ASKA library^25^. ASKA plasmids were recovered from the ASKA collection using a QIAprep Miniprep Kit (Qiagen), transformed into the corresponding *sgl* expression strain and plated on LB + K + C agar. Transformants were verified by Sanger sequencing.

Plate reader assays for validations were performed as follows. Strains were inoculated into LB + K + C overnight. Cells were diluted 50× into LB + K + C media and allowed to grow at 37 °C and 180 r.p.m. to OD_600_ = 0.5. Cells were then transferred to a 96-well plate (Corning, 3904) and induced with varying levels of aTc ranging from 0 ng ml^−1^ to 250 ng ml^−1^ for *sgl* expression and varying levels of IPTG ranging from 0 µM to 200 µM for ASKA gene expression. Sgl lysis progressed in Tecan Infinite F200 readers with orbital shaking and OD_600_ readings every 10 minutes for 3–5 hours at 37 °C. Strains with unsuppressed lysis phenotypes typically had visible lysis after ~90 minutes.

### Suppression by heterologous flippase genes

Strain TB28 *E. coli* was co-transformed with plasmids carrying inducible *sgl* genes (*sgl*^*M*^, *sgl*^*PP7*^ and *GFP-sgl*^*PP7*^) and compatible plasmids expressing MurJ orthologs (MurJ_TA_^35^ and AmJ^36^) and selected on LB–Ampicliin–Spectinomycin–IPTG (100 μM) agar plates. The transformants were grown overnight at 37 °C with the same selective media, and, on the following day, 1:200 dilutions of the overnights were added to 25 ml of LB with appropriate antibiotics and IPTG (100 μM) in a 250-ml flask and grown at 37 °C in an orbital shaker (New Brunswick Scientific gyrotory water bath shaker, G76) at 250 r.p.m. The cultures were induced with 0.4% w/v l-arabinose (Sigma-Aldrich) at OD_550_ of ~0.2. The growth curves were plotted using RStudio version 1.3.1073 and Inkscape 1.0.

### Isolation of GFP-Sgl^PP7^-resistant mutants

Cultures of XL1-Blue pBAD24-*gfp-sgl*^*PP7*^*-lacZ*_*α*_ were grown overnight at 37 °C with aeration. To perform the Sgl screen/selection, 100 μl of overnight culture was mixed with 400 μl of LB and plated on LB–Arabinose–Ampicllin–IPTG–X-gal agar plates (100 mm). After overnight incubation at 37 °C, blue colonies were picked and purified on the same selection media. The Sgl^PP7^-resistant colonies were grown overnight, and both genomic (Qiagen QIAamp DNA Micro Kit) and plasmid (Qiagen Miniprep Kit) DNA were extracted. To rule out possible mutations in the lysis gene, the plasmid was sequenced with primers KC30 and KC31. The *murJ* locus in the gDNA of the Sgl^PP7^-resistant mutants was amplified by PCR using Phusion (New England Biolabs) with the primers KC230 and KC234. The amplified PCR product was gel-purified and sequenced with the primers KC230, KC231, KC232, KC233 and KC234.

### Reporting summary

Further information on research design is available in the Nature Portfolio Reporting Summary linked to this article.

## Online content

Any methods, additional references, Nature Portfolio reporting summaries, source data, extended data, supplementary information, acknowledgements, peer review information; details of author contributions and competing interests; and statements of data and code availability are available at 10.1038/s41589-023-01269-7.

## Supplementary information

Supplementary Information Supplementary Figs. 1–6 and Supplementary Tables 1 and 2.

Reporting Summary

Supplementary Data 1 Overview of all library experiments and time 0s used in this study. ‘Set’ and ‘Index’ values are used to identify raw next-generation sequencing reads.

Supplementary Data 2 Gene-level Dub-seq library coverage for all reported libraries.

Supplementary Data 3 Barcode-level Illumina reads counts for all reported library screens.

Supplementary Data 4 Barcode-level Dub-seq scores (f-scores) for all reported library screens.

Supplementary Data 5 Gene-level Dub-seq scores (g-scores) for all reported library screens.

Supplementary Data 6 Description of strains and plasmids used in this study. An overview of libraries is given in Supplementary Table 1.

Supplementary Data 7 Primers and genes used in this study.

Supplementary Data 8 Source data information for Supplementary Figs. 1–6.

## Data Availability

Sequencing data have been uploaded to the Sequence Read Archive under BioProject accession number PRJNA800467. Complete data from all experiments (read counts per barcode, fragment scores and gene scores) are available here at 10.6084/m9.figshare.21714296.v2. MurJ_EC_ structure was downloaded from the Research Collaboratory for Structural Bioinformatics Protein Data Bank (6CC4). The data underlying Figs. 1c,d, 2b and 4b and Supplementary Figs. 1–6 are provided as source data. Plasmids and strains are available from the corresponding author upon reasonable request. Source data are provided with this paper.

## Source data

Source Data Fig. 1 Source data to generate Fig. 1c,d.
Source Data Fig. 2 Source data to generate Fig. 2b.
Source Data Fig. 3 Source data to generate Fig. 3.
Source Data Fig. 4 Source data to generate Fig. 4.

## Code availability

Code for processing and analyzing Dub-seq data is available at https://github.com/psnovichkov/DubSeq. We used the Dub-seq viewer tool from the Dub-seq Python library (https://github.com/psnovichkov/DubSeq) to generate regions of the *E. coli* chromosome covering fragments presented in Figs. 1c and 3a,c and Supplementary Figs. 1–6 in the landscape mode.
