# Supplementary material for: Multicopy suppressor screens reveal convergent evolution of single-gene lysis proteins
Source: Nat Chem Biol. 2023 Feb 20;19(6):759–66. doi: 10.1038/s41589-023-01269-7 (PMC10229422; doi:10.1038/s41589-023-01269-7)
Supplement: Supplementary file 1 — Supplementary Figs. 1–6 and Supplementary Tables 1 and 2. [file 41589_2023_1269_MOESM1_ESM.pdf]

nature chemical biology

Article

https://doi.org/10.1038/s41589-023-01269-7

Multicopy suppressor screens reveal convergent evolution of single-gene lysis proteins

In the format provided by the authors and unedited

Supplementary information Contents:

Supplementary Table 1: Overview of Dub-seq library composition for all libraries employed in this study.

Supplementary Table 2: Taxonomy information for different single strand RNA phages referenced in this work.

Supplementary Fig. 1: Detailed Dub-seq suppressor screening results against Sgl^{M}.

Supplementary Fig. 2: Detailed Dub-seq suppressor screening results against Sgl^{Hgal1}.

Supplementary Fig. 3: Detailed Dub-seq suppressor screening results against Sgl^{Ku1}.

Supplementary Fig. 4: Detailed Dub-seq suppressor screening results against Sgl^{MS2}.

Supplementary Fig. 5: Detailed Dub-seq suppressor screening results against Sgl^{PP7}.

Supplementary Fig. 6: Detailed Dub-seq suppressor screening results against Sgl^{PRR1}.

Supplementary Fig. 7: Sequence alignment of *Fiersviridae* lysis proteins.

Source data files

Source data file for each figure in the main text is submitted as a seaparate file

Supplementary Table 1. DubSeq suppressor libraries employed in this study. The source genome for all libraries is BW25113 with 4509 potential genes for inclusion in Dub-seq experiments. The original pFAB5516 Dub-seq library is included as a reference.

Library	Sgl Name? [1]	Established Sgl Target?	Source Phage Genome	Number Fragments	Median Fragment Length (bp)	Genes Covered?	Reference
pFAB5516	N/A	N/A	N/A	27778	2514	4336	PMID: 30659179
sgl^{Hgal1} + pFAB5516	Sgl^{Hgal1}	No	NC_019922	15639	2439	3736	This study
sgl^{Ku1} + pFAB5516	Sgl^{Ku1}	No	AF227250.1	12916	2426	4042	This study
sgl^{M} + pFAB5516	LysM, Sgl^{M}	MurJ	NC_019707	17007	2455	3846	PMID: 28894177; This study
sgl^{MS2} + pFAB5516	L, Sgl^{MS2}	No	NC_001417	16208	2443	3802	This study
sgl^{PRR1} + pFAB5516	Sgl^{PRR1}	No	NC_008294	18477	2453	3924	This study
sgl^{PP7} + pFAB5516	Sgl^{PP7}	No	NC_001628	20940	2463	3968	This study

[1] Recent identification and expansion of Sgls necessitates a more systematic nomenclature for these proteins, but some have less informative common nomenclature. Here, we use a format consistent with Chamakura and Young 2020 (PMID:33075663). For instance, the Sgl from phage M is Sgl^{M}.

***Supplementary Table 2. Taxonomy information for different single strand RNA phages referenced in this work*.**

Phage	Family	Genus	Species	txid
Hgal1	*Fiersviridae*	*Hagavirus*	*Hagavirus psychrophilum*	1206300
Ku1	*Fiersviridae*	*Emesvirus*	*Emesvirus japonicum*	12021
M	*Fiersviridae*	*Empivirus*	*Empivirus allolyticum*	1235640
MS2	*Fiersviridae*	*Emesvirus*	*Emesvirus zinderi*	12022
PP7	*Fiersviridae*	*Pepevirus*	*Pepevirus rubrum*	12023
PRR1	*Fiersviridae*	*Perrunavirus*	*Perrunavirus olsenii*	12024

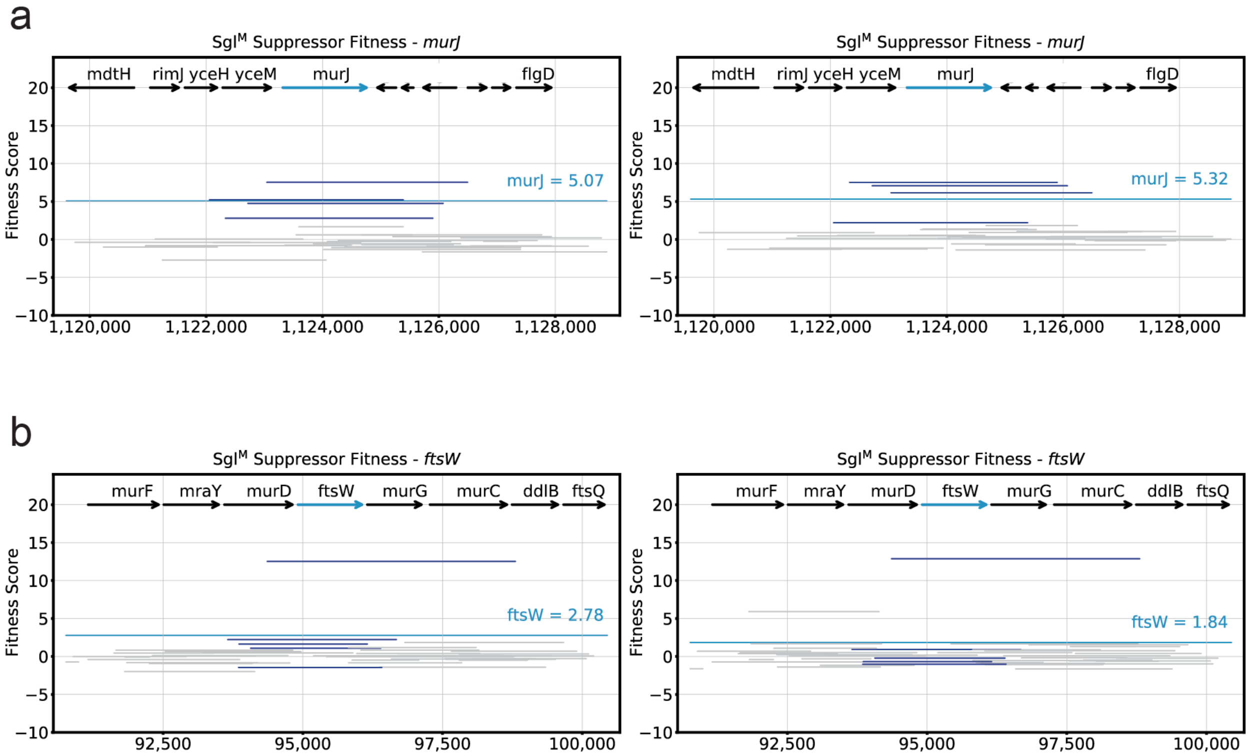

Supplementary Fig. 1: Detailed Dub-seq suppressor screening results against Sgl^{M}. (a-b) Dub-seq plots for suppressor screening against Sgl^{M} zoomed in on a gene of interest with experimental replicates shown side-by-side. Genes of interest are significant hits shown in Fig. 2 or candidate multi-gene multi-copy suppressors. Black arrows represent tracked ORFs during analysis. Teal arrows represent the gene of interest. Dark blue lines represent Dub-seq fragments covering the gene of interest with scores shown as *fscores*. Gray lines represent Dub-seq fragments that do not entirely cover the gene of interest with scores shown as *fscores*. Teal lines represent the gene *gscore* of the gene of interest.

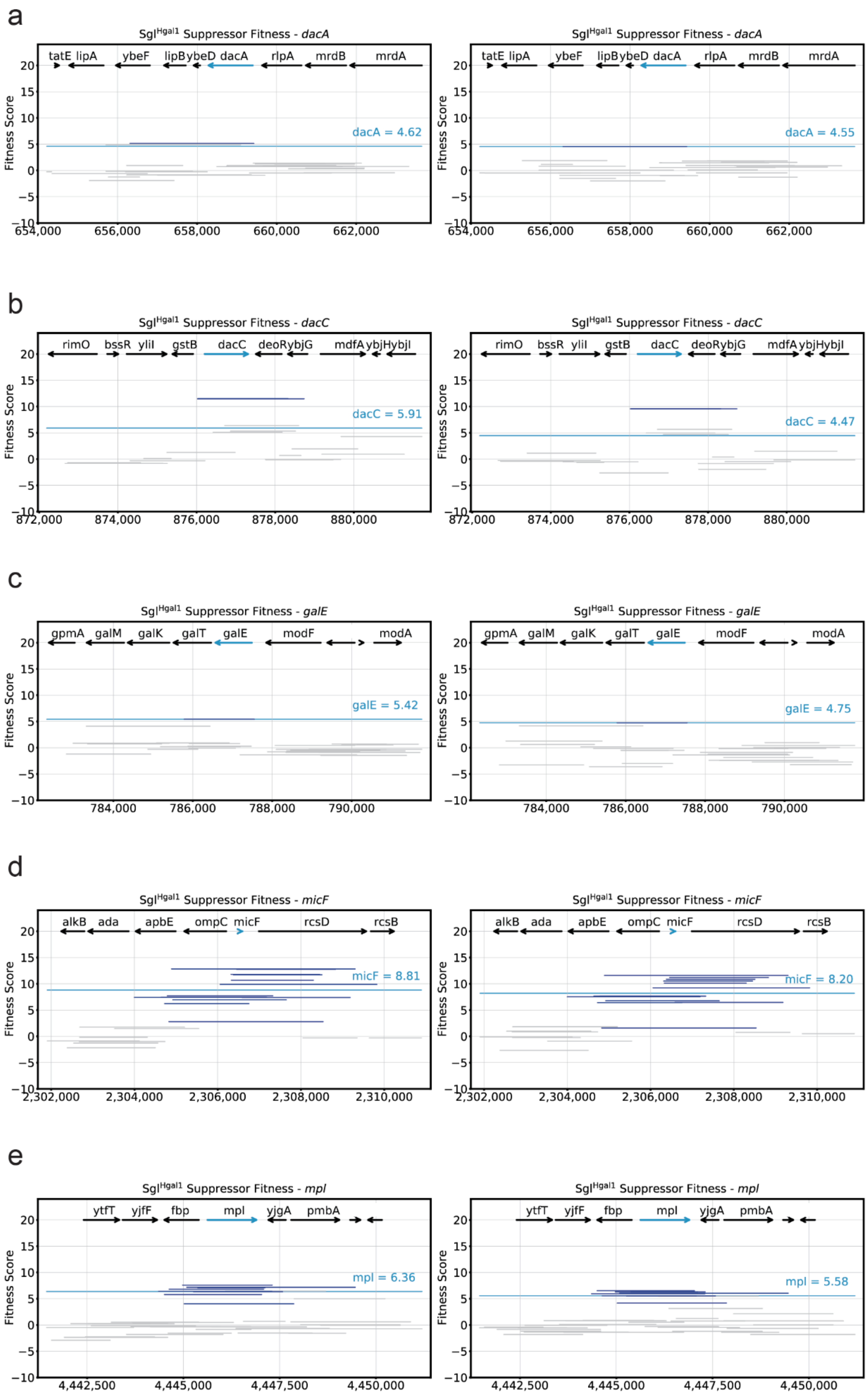
a
Sgl^{Hgal1} Suppressor Fitness - *dacA*
tatE lipA ybeF lipB ybeD dacA rlpA mrdB mrdA
dacA = 4.62
dacA = 4.55
Fitness Score
654,000 656,000 658,000 660,000 662,000
b
Sgl^{Hgal1} Suppressor Fitness - *dacC*
rimO bssR yliI gstB dacC deoR ybjG mdfA ybjH ybjI
dacC = 5.91
dacC = 4.47
872,000 874,000 876,000 878,000 880,000
c
Sgl^{Hgal1} Suppressor Fitness - *galE*
gpmA galM galK galT galE modF modA
galE = 5.42
galE = 4.75
784,000 786,000 788,000 790,000
d
Sgl^{Hgal1} Suppressor Fitness - *micF*
alkB ada apbE ompC micF rcsD rcsB
micF = 8.81
micF = 8.20
2,302,000 2,304,000 2,306,000 2,308,000 2,310,000
e
Sgl^{Hgal1} Suppressor Fitness - *mpl*
ytfT yjfF fbp mpl yjgA pmbA
mpl = 6.36
mpl = 5.58
4,442,500 4,445,000 4,447,500 4,450,000

63

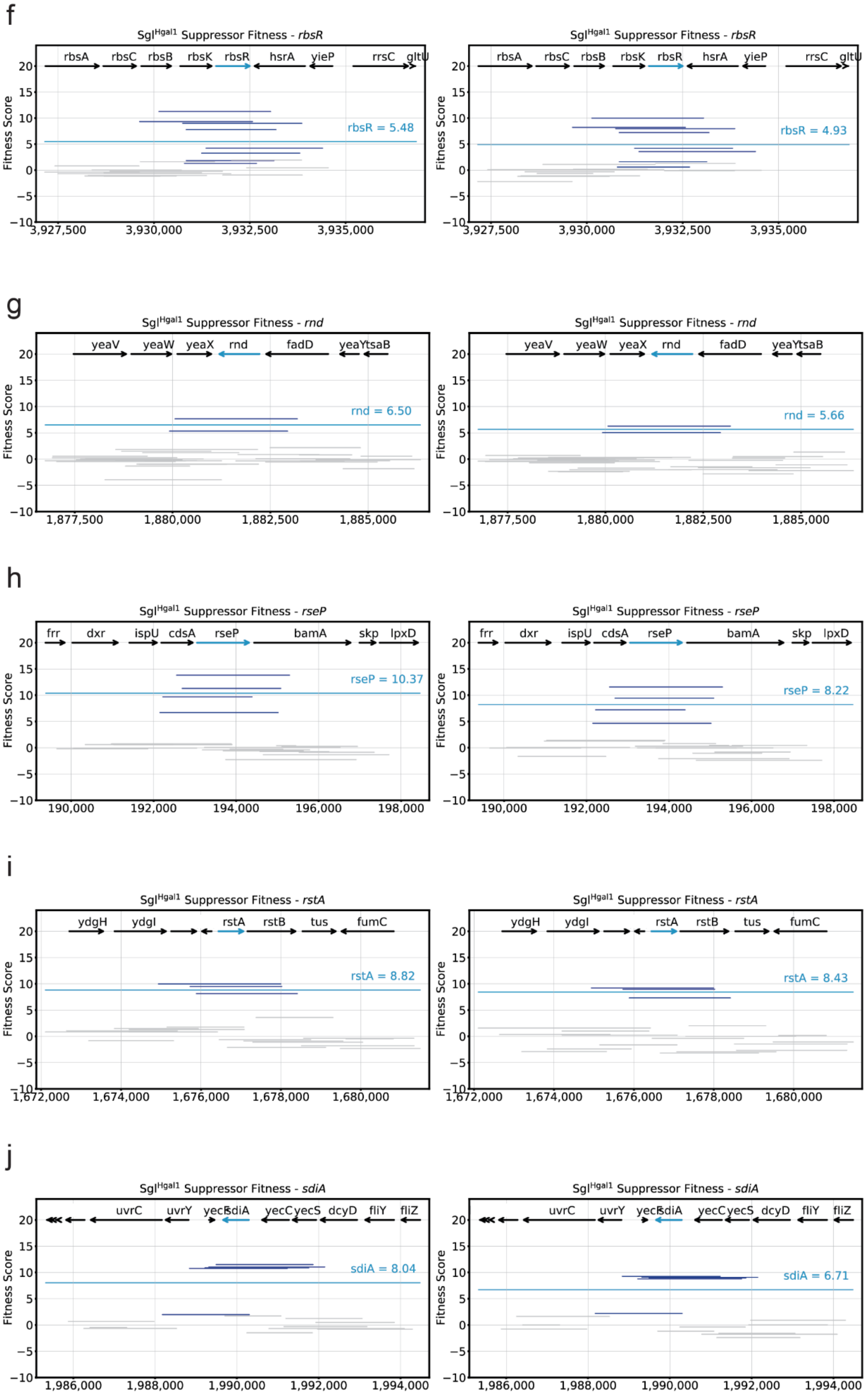
f
Sgl^{Hgal1} Suppressor Fitness - *rbsR*
rbsA rbsC rbsB rbsK rbsR hsrA yieP rrsC gltU
rbsR = 5.48
rbsR = 4.93
Fitness Score
3,927,500 3,930,000 3,932,500 3,935,000
g
Sgl^{Hgal1} Suppressor Fitness - *rnd*
yeaV yeaW yeaX rnd fadD yeaYtsaB
rnd = 6.50
rnd = 5.66
1,877,500 1,880,000 1,882,500 1,885,000
h
Sgl^{Hgal1} Suppressor Fitness - *rseP*
frr dxr ispU cdsA rseP bamA skp lpxD
rseP = 10.37
rseP = 8.22
190,000 192,000 194,000 196,000 198,000
i
Sgl^{Hgal1} Suppressor Fitness - *rstA*
ydgH ydgI rstA rstB tus fumC
rstA = 8.82
rstA = 8.43
1,672,000 1,674,000 1,676,000 1,678,000 1,680,000
j
Sgl^{Hgal1} Suppressor Fitness - *sdiA*
uvrC uvrY yecF sdiA yecC yecS dcyD fliY fliZ
sdiA = 8.04
sdiA = 6.71
1,986,000 1,988,000 1,990,000 1,992,000 1,994,000

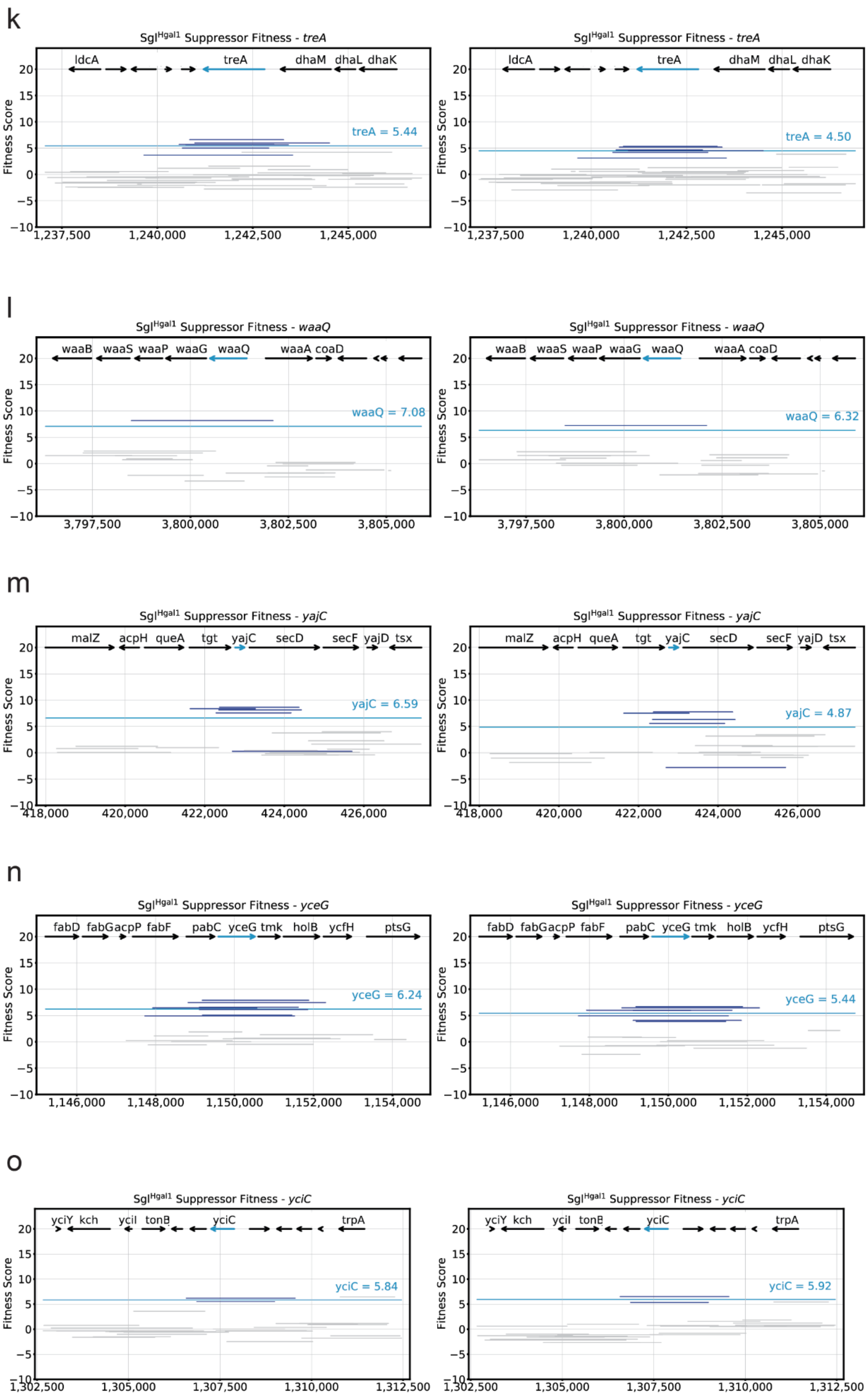
k
Sgl^{Hgal1} Suppressor Fitness - *treA*
ldcA
treA
dhaM
dhaL
dhaK
treA = 5.44
treA = 4.50
Fitness Score
1,237,500
1,240,000
1,242,500
1,245,000
l
Sgl^{Hgal1} Suppressor Fitness - *waaQ*
waaB
waaS
waaP
waaG
waaQ
waaA
coaD
waaQ = 7.08
waaQ = 6.32
Fitness Score
3,797,500
3,800,000
3,802,500
3,805,000
m
Sgl^{Hgal1} Suppressor Fitness - *yajC*
malZ
acpH
queA
tgt
yajC
secD
secF
yajD
tsx
yajC = 6.59
yajC = 4.87
Fitness Score
418,000
420,000
422,000
424,000
426,000
n
Sgl^{Hgal1} Suppressor Fitness - *yceG*
fabD
fabG
acpP
fabF
pabC
yceG
tmk
holB
ycfH
ptsG
yceG = 6.24
yceG = 5.44
Fitness Score
1,146,000
1,148,000
1,150,000
1,152,000
1,154,000
o
Sgl^{Hgal1} Suppressor Fitness - *yciC*
yciY
kch
yciI
tonB
yciC
trpA
yciC = 5.84
yciC = 5.92
Fitness Score
1,302,500
1,305,000
1,307,500
1,310,000
1,312,500

p

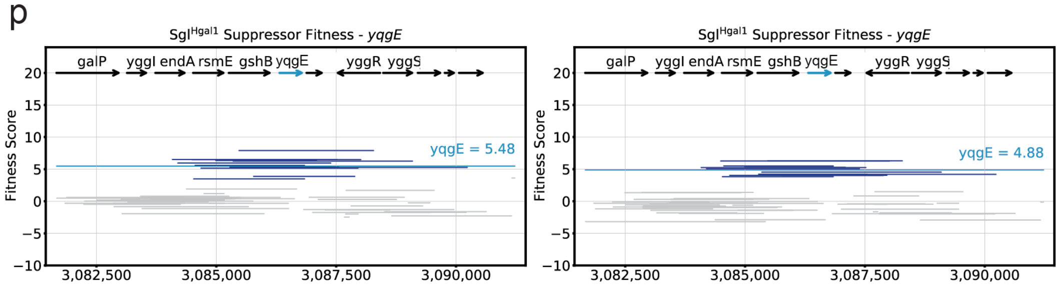

q

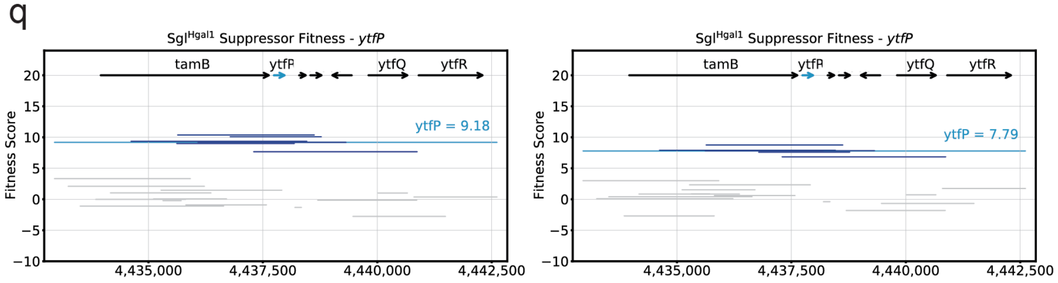

Supplementary Fig. 2: Detailed Dub-seq suppressor screening results against Sgl^{Hgal1}. (a-q) Dub-seq plots for suppressor screening against Sgl^{Hgal1} zoomed in on a gene of interest with experimental replicates shown side-by-side. Genes of interest are significant hits shown in Fig. 2 as candidate multi-gene multi-copy suppressors. Black arrows represent tracked ORFs during analysis. Teal arrows represent the gene of interest. Dark blue lines represent Dub-seq fragments covering the gene of interest with scores shown as *fscores*. Gray lines represent Dub-seq fragments that do not entirely cover the gene of interest with scores shown as *fscores*. Teal lines represent the *gscore* of the gene of interest.

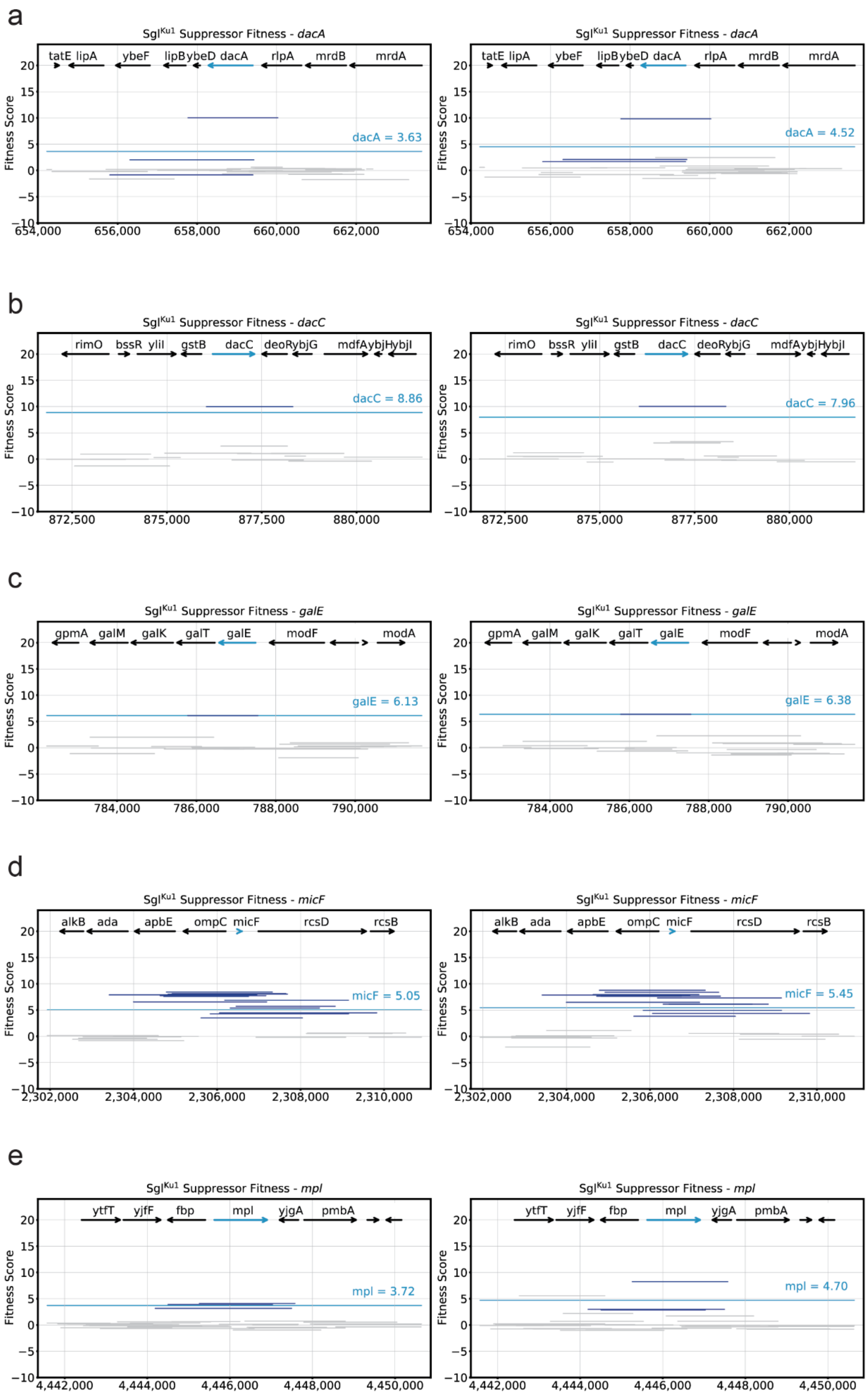
a
SglKu1 Suppressor Fitness - dacA
tatE lipA ybeF lipBybeD dacA rlpA mrdB mrdA
dacA = 3.63
dacA = 4.52
Fitness Score
654,000 656,000 658,000 660,000 662,000
b
SglKu1 Suppressor Fitness - dacC
rimO bssR yliI gstB dacC deoRybjG mdfAybjHybjI
dacC = 8.86
dacC = 7.96
Fitness Score
872,500 875,000 877,500 880,000
c
SglKu1 Suppressor Fitness - galE
gpmA galM galK galT galE modF modA
galE = 6.13
galE = 6.38
Fitness Score
784,000 786,000 788,000 790,000
d
SglKu1 Suppressor Fitness - micF
alkB ada apbE ompC micF rcsD rcsB
micF = 5.05
micF = 5.45
Fitness Score
2,302,000 2,304,000 2,306,000 2,308,000 2,310,000
e
SglKu1 Suppressor Fitness - mpl
ytfT yjfF fbp mpl yjgA pmbA
mpl = 3.72
mpl = 4.70
Fitness Score
4,442,000 4,444,000 4,446,000 4,448,000 4,450,000

f

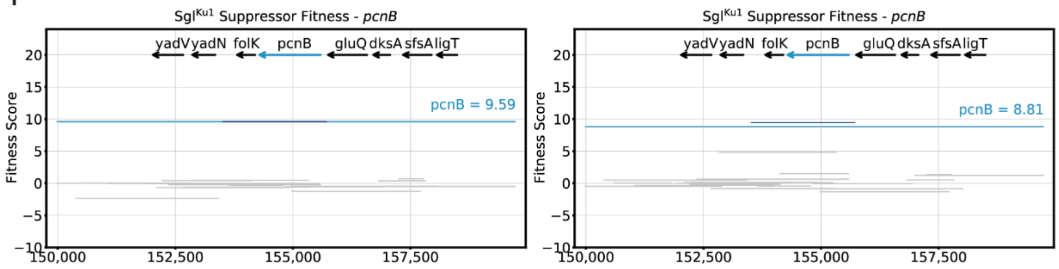

Sgl^{Ku1} Suppressor Fitness - *pcnB*
yadV yadN folK pcnB gluQ dksA sfsA ligT
pcnB = 9.59
Fitness Score
150,000
152,500
155,000
157,500
Sgl^{Ku1} Suppressor Fitness - *pcnB*
yadV yadN folK pcnB gluQ dksA sfsA ligT
pcnB = 8.81
Fitness Score
150,000
152,500
155,000
157,500

g

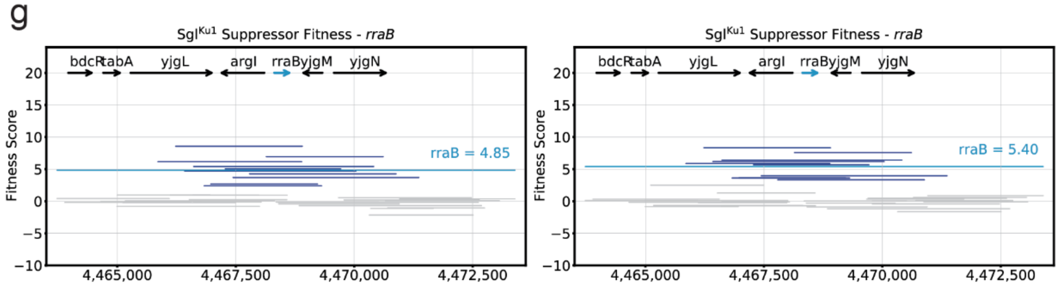

Sgl^{Ku1} Suppressor Fitness - *rraB*
bdcR tabA yjgL argI rraB yjgM yjgN
rraB = 4.85
Fitness Score
4,465,000
4,467,500
4,470,000
4,472,500
Sgl^{Ku1} Suppressor Fitness - *rraB*
bdcR tabA yjgL argI rraB yjgM yjgN
rraB = 5.40
Fitness Score
4,465,000
4,467,500
4,470,000
4,472,500

h

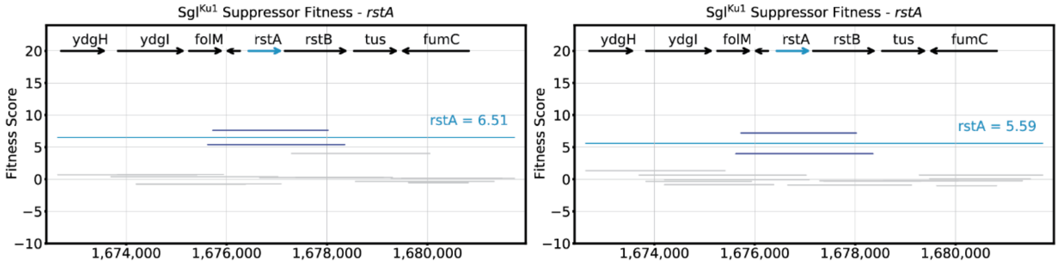

Sgl^{Ku1} Suppressor Fitness - *rstA*
ydgH ydgI folM rstA rstB tus fumC
rstA = 6.51
Fitness Score
1,674,000
1,676,000
1,678,000
1,680,000
Sgl^{Ku1} Suppressor Fitness - *rstA*
ydgH ydgI folM rstA rstB tus fumC
rstA = 5.59
Fitness Score
1,674,000
1,676,000
1,678,000
1,680,000

i

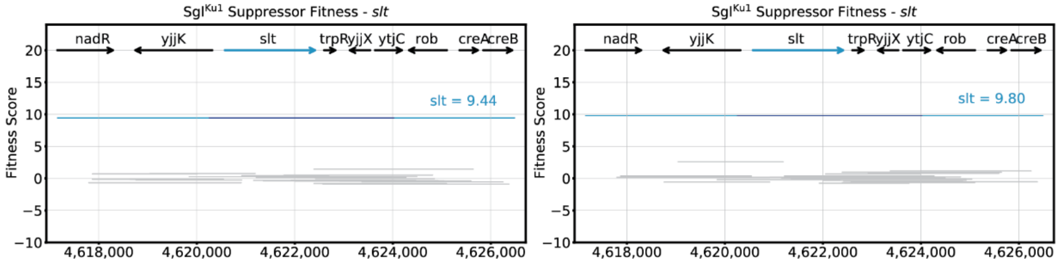

Sgl^{Ku1} Suppressor Fitness - *slt*
nadR yjjK slt trpR yjjX ytjC rob creA creB
slt = 9.44
Fitness Score
4,618,000
4,620,000
4,622,000
4,624,000
4,626,000
Sgl^{Ku1} Suppressor Fitness - *slt*
nadR yjjK slt trpR yjjX ytjC rob creA creB
slt = 9.80
Fitness Score
4,618,000
4,620,000
4,622,000
4,624,000
4,626,000

j

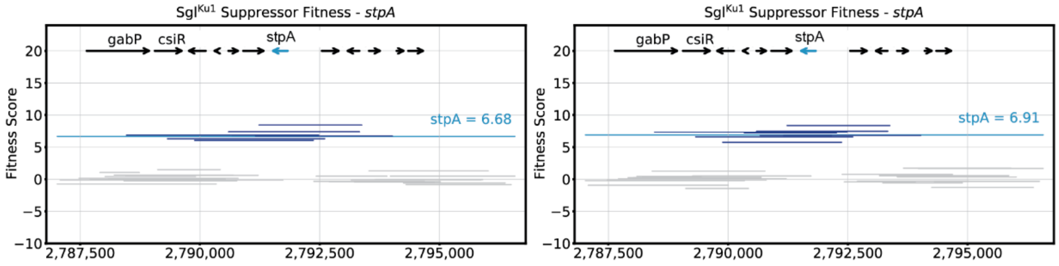

Sgl^{Ku1} Suppressor Fitness - *stpA*
gabP csiR stpA
stpA = 6.68
Fitness Score
2,787,500
2,790,000
2,792,500
2,795,000
Sgl^{Ku1} Suppressor Fitness - *stpA*
gabP csiR stpA
stpA = 6.91
Fitness Score
2,787,500
2,790,000
2,792,500
2,795,000

k

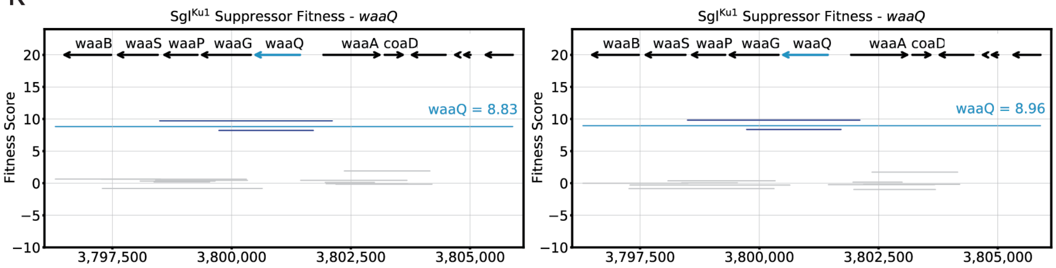

l

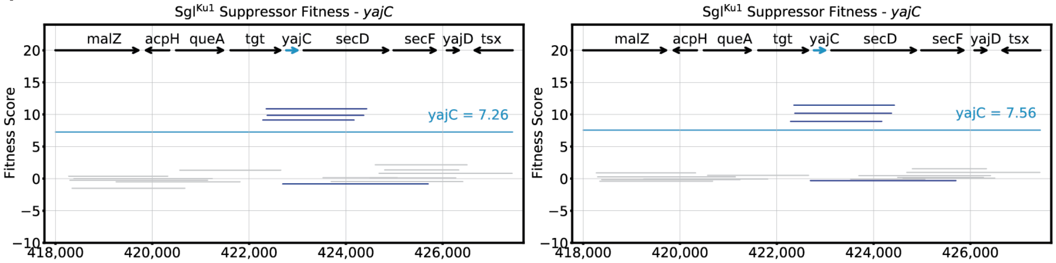

m

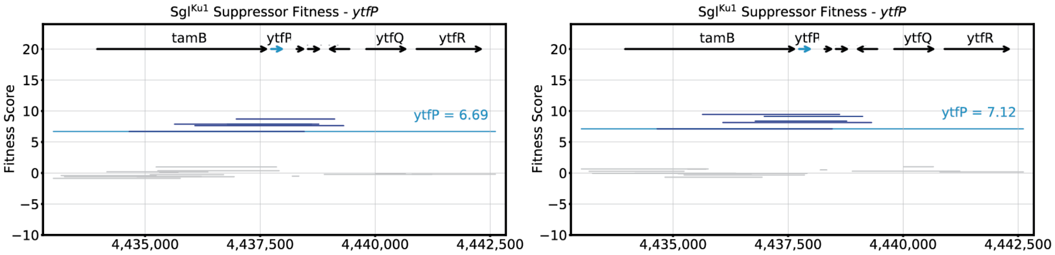

Supplementary Fig. 3: Detailed Dub-seq suppressor screening results against Sgl^{Ku1}. (a-m) Dub-seq plots for suppressor screening against Sgl^{Ku1} zoomed in on a gene of interest with experimental replicates shown side-by-side. Genes of interest are significant hits shown in Fig. 2 or candidate multi-gene multi-copy suppressors. Black arrows represent tracked ORFs during analysis. Teal arrows represent the gene of interest. Dark blue lines represent Dub-seq fragments covering the gene of interest with scores shown as *fscores*. Gray lines represent Dub-seq fragments that do not entirely cover the gene of interest with scores shown as *fscores*. Teal lines represent the *gscore* of the gene of interest.

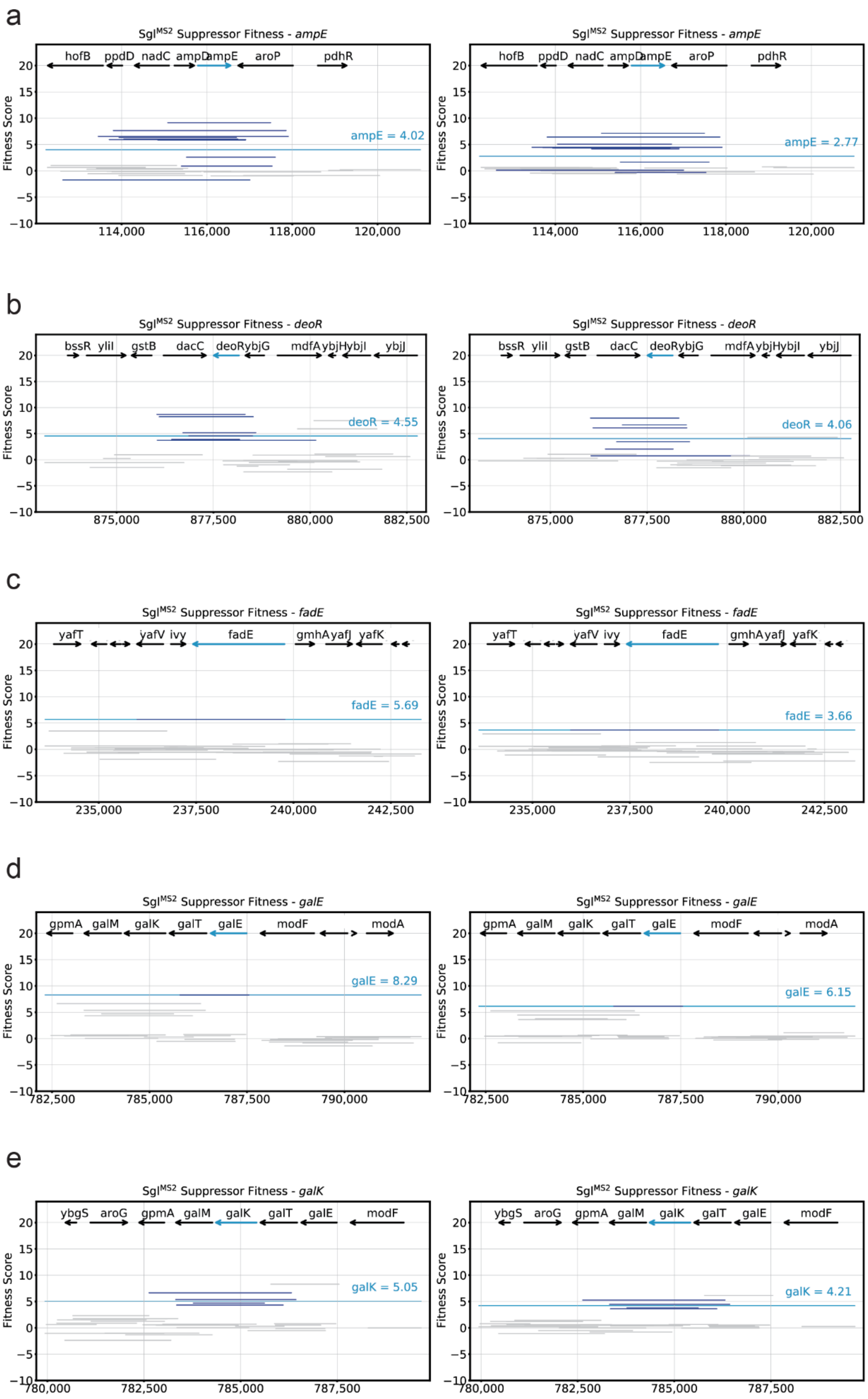
a
SglMS2 Suppressor Fitness - ampE
hofB ppdD nadC ampD ampE aroP pdhR
ampE = 4.02
ampE = 2.77
Fitness Score
114,000 116,000 118,000 120,000
b
SglMS2 Suppressor Fitness - deoR
bssR yliI gstB dacC deoR ybjG mdfA ybjH ybjI ybjJ
deoR = 4.55
deoR = 4.06
875,000 877,500 880,000 882,500
c
SglMS2 Suppressor Fitness - fadE
yafT yafV ivy fadE gmhA yafJ yafK
fadE = 5.69
fadE = 3.66
235,000 237,500 240,000 242,500
d
SglMS2 Suppressor Fitness - galE
gpmA galM galK galT galE modF modA
galE = 8.29
galE = 6.15
782,500 785,000 787,500 790,000
e
SglMS2 Suppressor Fitness - galK
ybgS aroG gpmA galM galK galT galE modF
galK = 5.05
galK = 4.21
780,000 782,500 785,000 787,500

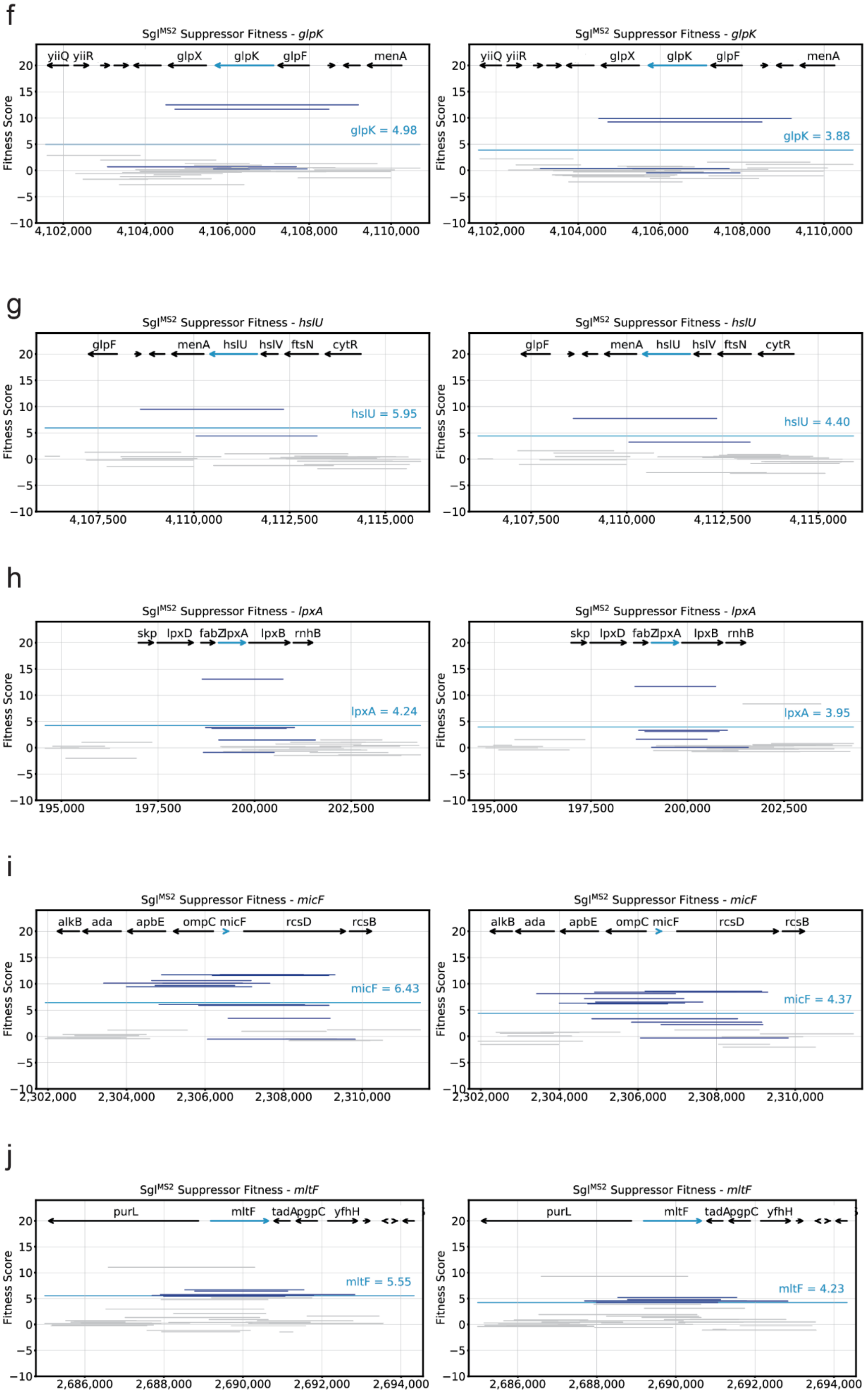

f
Sgl^MS2 Suppressor Fitness - glpK
yiiQ yiiR
glpX
glpK
glpF
menA
glpK = 4.98
glpK = 3.88
Fitness Score
4,102,000
4,104,000
4,106,000
4,108,000
4,110,000
g
Sgl^MS2 Suppressor Fitness - hslU
glpF
menA
hslU
hslV
ftsN
cytR
hslU = 5.95
hslU = 4.40
4,107,500
4,110,000
4,112,500
4,115,000
h
Sgl^MS2 Suppressor Fitness - lpxA
skp
lpxD
fabZ
lpxA
lpxB
rnhB
lpxA = 4.24
lpxA = 3.95
195,000
197,500
200,000
202,500
i
Sgl^MS2 Suppressor Fitness - micF
alkB
ada
apbE
ompC micF
rcsD
rcsB
micF = 6.43
micF = 4.37
2,302,000
2,304,000
2,306,000
2,308,000
2,310,000
j
Sgl^MS2 Suppressor Fitness - mltF
purL
mltF
tadA
pgpC
yfhH
mltF = 5.55
mltF = 4.23
2,686,000
2,688,000
2,690,000
2,692,000
2,694,000

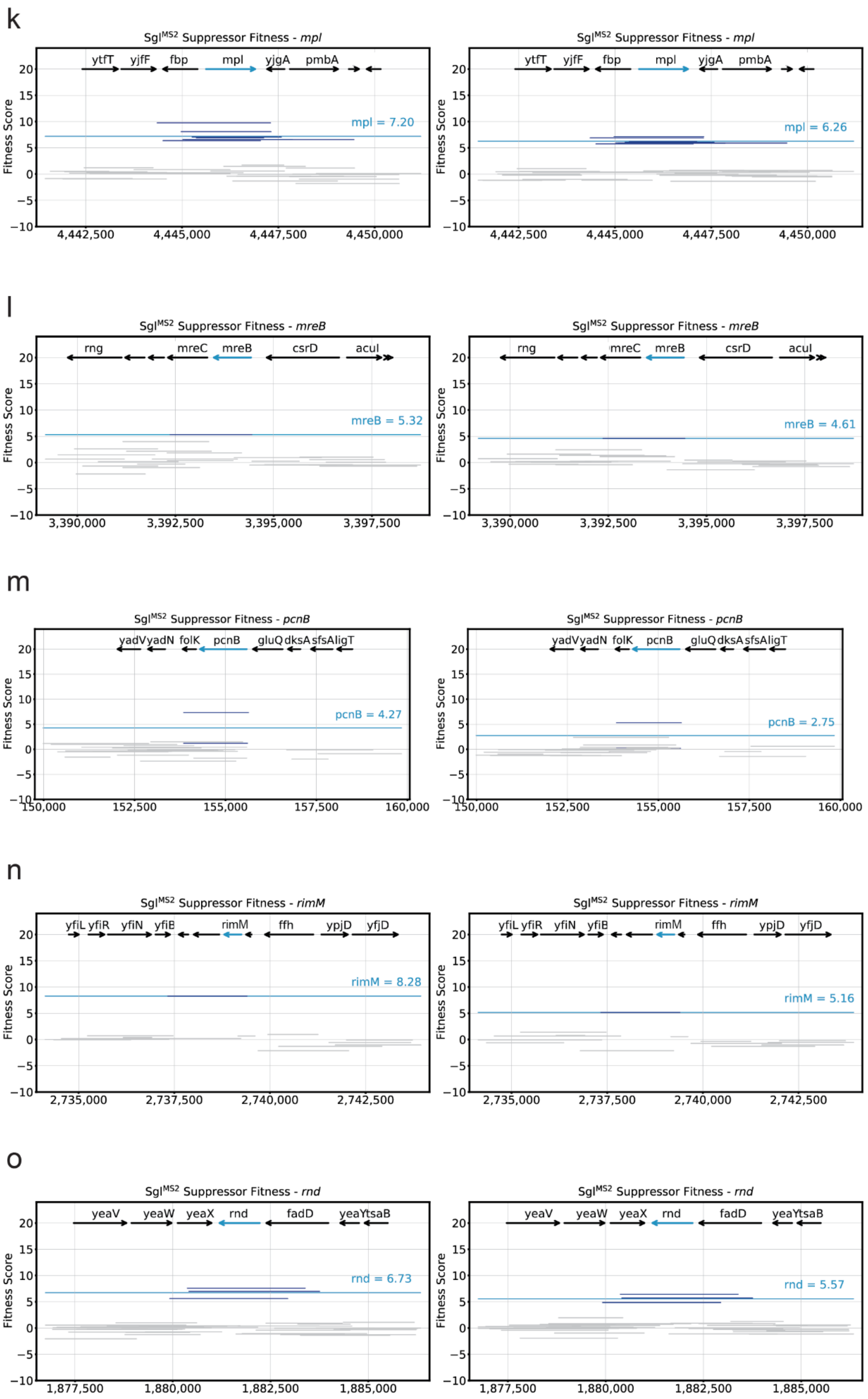
k
Sgl^{MS2} Suppressor Fitness - *mpl*
ytfT yjfF fbp mpl yjgA pmbA
mpl = 7.20
mpl = 6.26
Fitness Score
4,442,500 4,445,000 4,447,500 4,450,000
l
Sgl^{MS2} Suppressor Fitness - *mreB*
rng mreC mreB csrD acuI
mreB = 5.32
mreB = 4.61
3,390,000 3,392,500 3,395,000 3,397,500
m
Sgl^{MS2} Suppressor Fitness - *pcnB*
yadV yadN folK pcnB gluQ dksA sfsA ligT
pcnB = 4.27
pcnB = 2.75
150,000 152,500 155,000 157,500 160,000
n
Sgl^{MS2} Suppressor Fitness - *rimM*
yfiL yfiR yfiN yfiB rimM ffh ypjD yfjD
rimM = 8.28
rimM = 5.16
2,735,000 2,737,500 2,740,000 2,742,500
o
Sgl^{MS2} Suppressor Fitness - *rnd*
yeaV yeaW yeaX rnd fadD yeaY tsaB
rnd = 6.73
rnd = 5.57
1,877,500 1,880,000 1,882,500 1,885,000

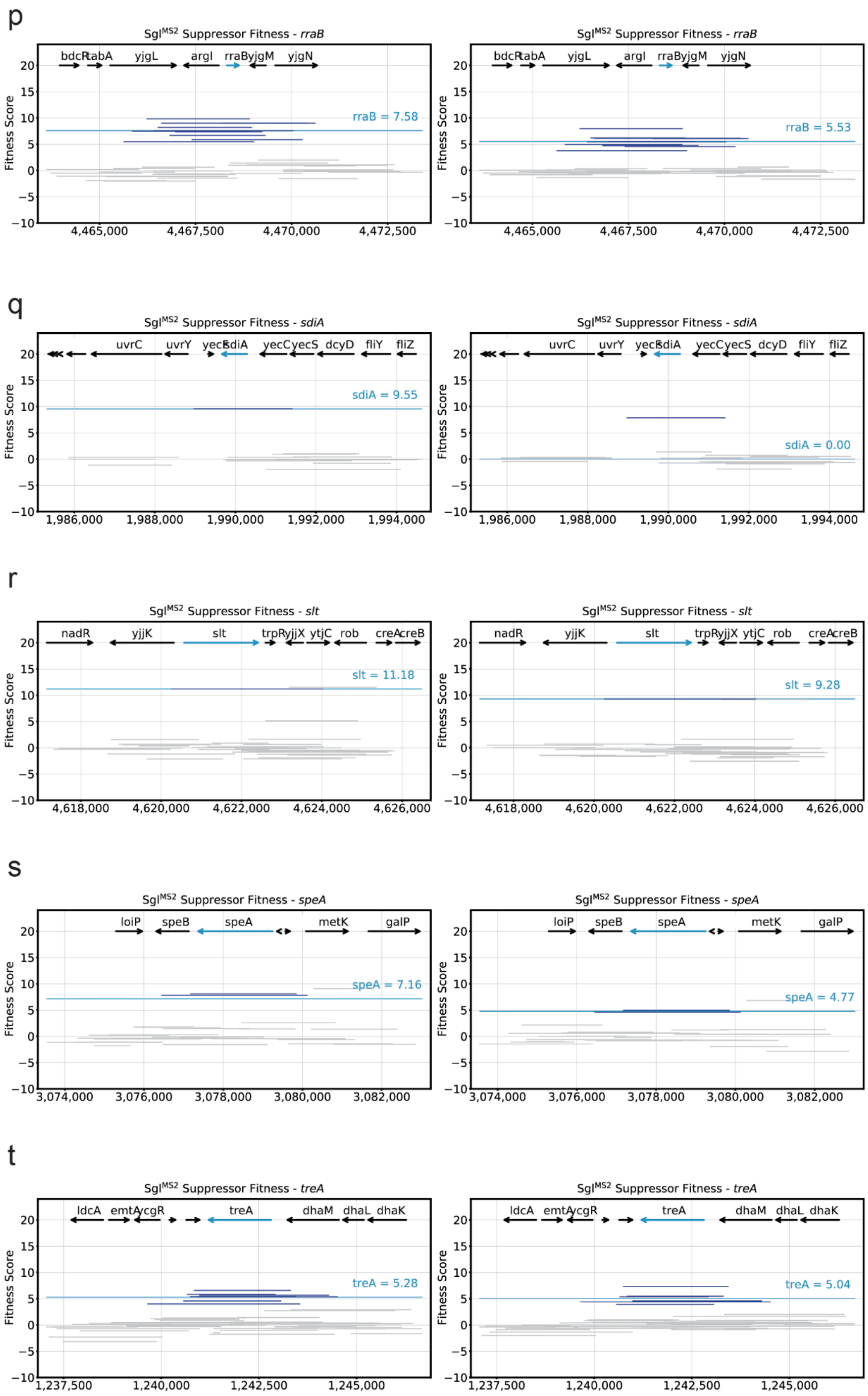
p
Sgl^{MS2} Suppressor Fitness - rraB
bdcR tabA yjgL argI rraB yjgM yjgN
rraB = 7.58
rraB = 5.53
Fitness Score
4,465,000 4,467,500 4,470,000 4,472,500
q
Sgl^{MS2} Suppressor Fitness - sdiA
uvrC uvrY yecF sdiA yecC yecS dcyD fliY fliZ
sdiA = 9.55
sdiA = 0.00
1,986,000 1,988,000 1,990,000 1,992,000 1,994,000
r
Sgl^{MS2} Suppressor Fitness - slt
nadR yjjK slt trpR yjjX ytjC rob creA creB
slt = 11.18
slt = 9.28
4,618,000 4,620,000 4,622,000 4,624,000 4,626,000
s
Sgl^{MS2} Suppressor Fitness - speA
loiP speB speA metK galP
speA = 7.16
speA = 4.77
3,074,000 3,076,000 3,078,000 3,080,000 3,082,000
t
Sgl^{MS2} Suppressor Fitness - treA
ldcA emtA ycgR treA dhaM dhaL dhaK
treA = 5.28
treA = 5.04
1,237,500 1,240,000 1,242,500 1,245,000

u

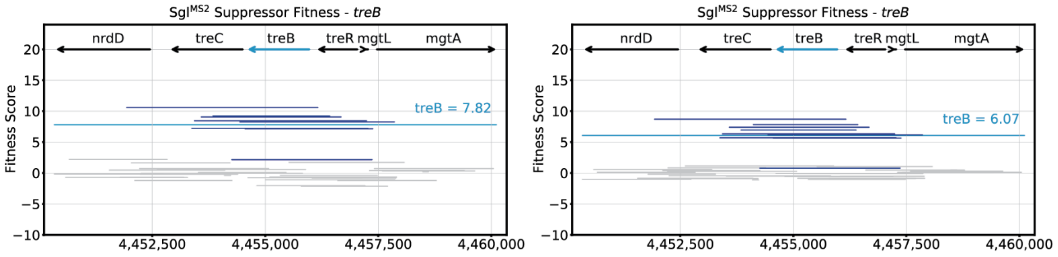

v

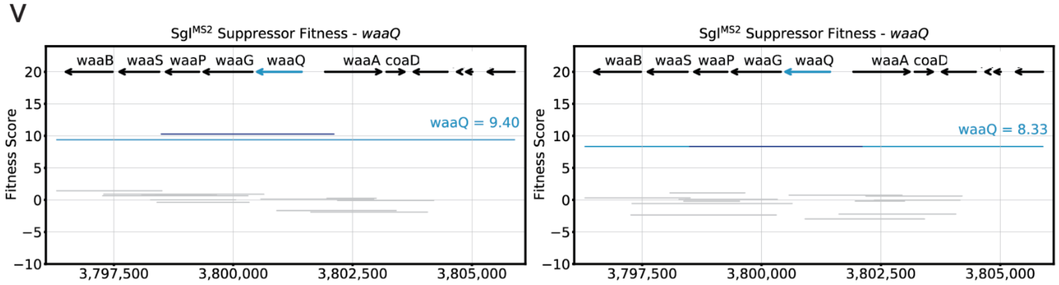

w

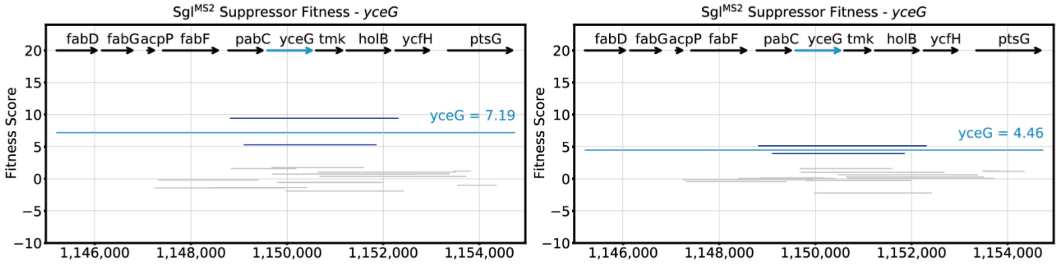

x

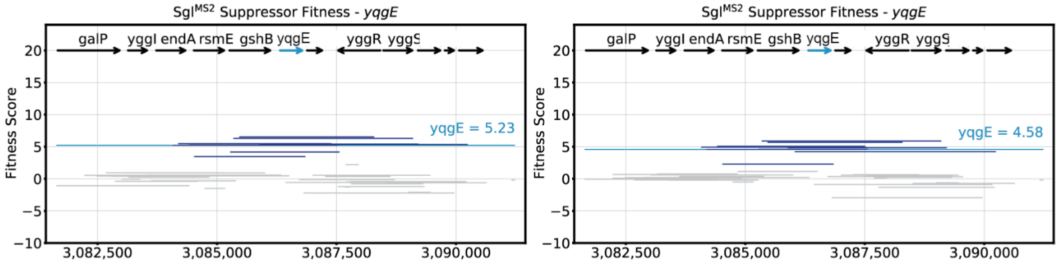

Supplementary Fig. 4: Detailed Dub-seq suppressor screening results against Sgl^{MS2} (a-x) Dub-seq plots for suppressor screening against Sgl^{MS2} zoomed in on a gene of interest with experimental replicates shown side-by-side. Genes of interest are significant hits shown in Fig. 2 or candidate multi-gene multi-copy suppressors. Black arrows represent tracked ORFs during analysis. Teal arrows represent the gene of interest. Dark blue lines represent Dub-seq fragments covering the gene of interest with scores shown as *fscores*. Gray lines represent Dub-seq fragments that do not entirely cover the gene of interest with scores shown as *fscores*. Teal lines represent the *gscore* of the gene of interest.

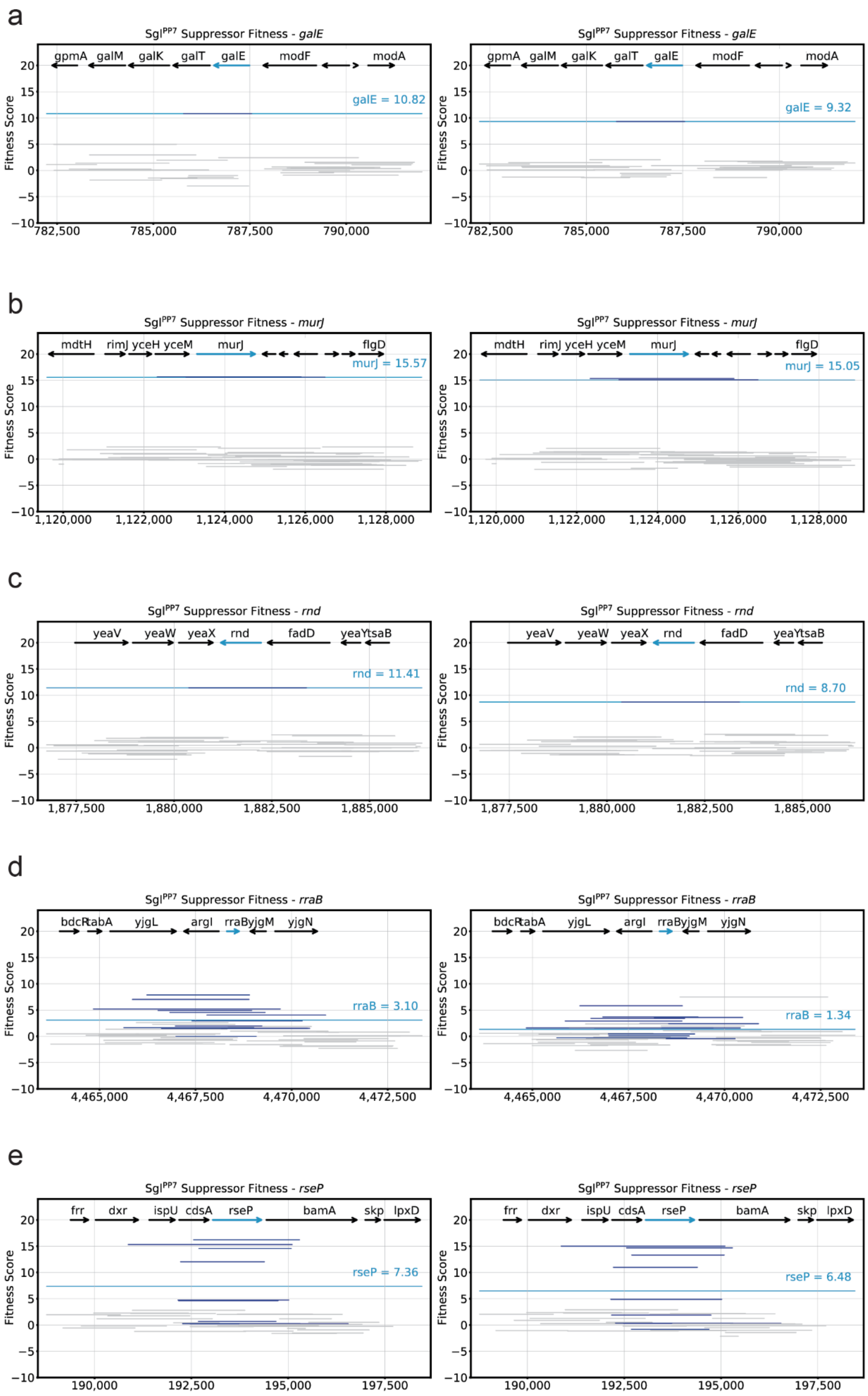
a
Sgl^{PP7} Suppressor Fitness - *galE*
gpmA galM galK galT galE modF modA
galE = 10.82
galE = 9.32
Fitness Score
782,500 785,000 787,500 790,000
b
Sgl^{PP7} Suppressor Fitness - *murJ*
mdtH rimJ yceH yceM murJ flgD
murJ = 15.57
murJ = 15.05
Fitness Score
1,120,000 1,122,000 1,124,000 1,126,000 1,128,000
c
Sgl^{PP7} Suppressor Fitness - *rnd*
yeaV yeaW yeaX rnd fadD yeaYtsaB
rnd = 11.41
rnd = 8.70
Fitness Score
1,877,500 1,880,000 1,882,500 1,885,000
d
Sgl^{PP7} Suppressor Fitness - *rraB*
bdcRtabA yjgL argI rraByjgM yjgN
rraB = 3.10
rraB = 1.34
Fitness Score
4,465,000 4,467,500 4,470,000 4,472,500
e
Sgl^{PP7} Suppressor Fitness - *rseP*
frr dxr ispU cdsA rseP bamA skp lpxD
rseP = 7.36
rseP = 6.48
Fitness Score
190,000 192,500 195,000 197,500

f

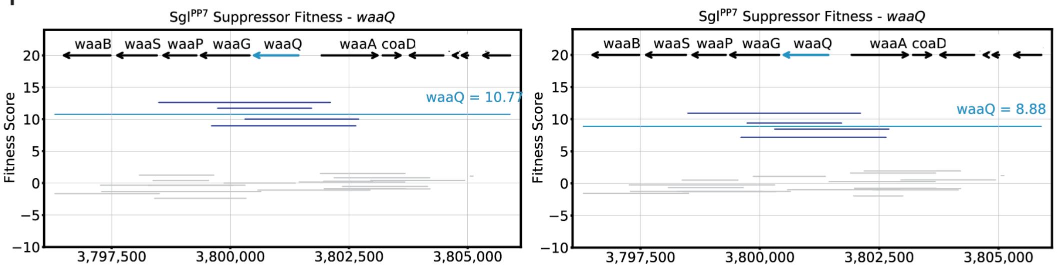

Supplementary Fig. 5: Detailed Dub-seq suppressor screening results against Sgl^{PP7}. (a-f) Dub-seq plots for suppressor screening against Sgl^{PP7} zoomed in on a gene of interest with experimental replicates shown side-by-side. Genes of interest are significant hits shown in Fig. 2 or candidate multi-gene multi-copy suppressors. Black arrows represent tracked ORFs during analysis. Teal arrows represent the gene of interest. Dark blue lines represent Dub-seq fragments covering the gene of interest with scores shown as *fscores*. Gray lines represent Dub-seq fragments that do not entirely cover the gene of interest with scores shown as *fscores*. Teal lines represent the *gscore* of the gene of interest.

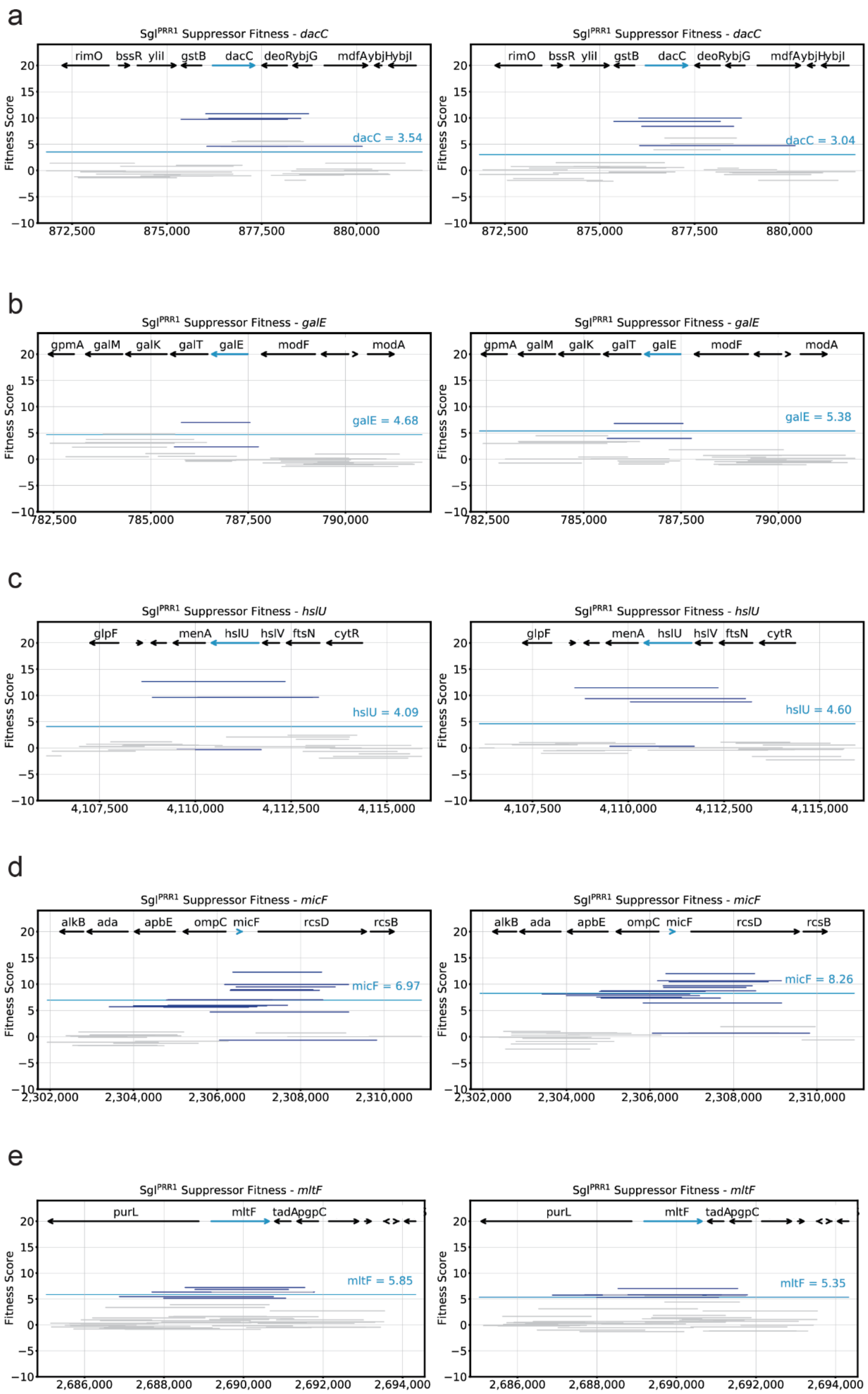
a
Sgl^PRR1 Suppressor Fitness - dacC
rimO bssR yliI gstB dacC deoRybjG mdfAybjHybjI
dacC = 3.54
dacC = 3.04
Fitness Score
872,500 875,000 877,500 880,000
b
Sgl^PRR1 Suppressor Fitness - galE
gpmA galM galK galT galE modF modA
galE = 4.68
galE = 5.38
782,500 785,000 787,500 790,000
c
Sgl^PRR1 Suppressor Fitness - hslU
glpF menA hslU hslV ftsN cytR
hslU = 4.09
hslU = 4.60
4,107,500 4,110,000 4,112,500 4,115,000
d
Sgl^PRR1 Suppressor Fitness - micF
alkB ada apbE ompC micF rcsD rcsB
micF = 6.97
micF = 8.26
2,302,000 2,304,000 2,306,000 2,308,000 2,310,000
e
Sgl^PRR1 Suppressor Fitness - mltF
purL mltF tadApgpC
mltF = 5.85
mltF = 5.35
2,686,000 2,688,000 2,690,000 2,692,000 2,694,000

113

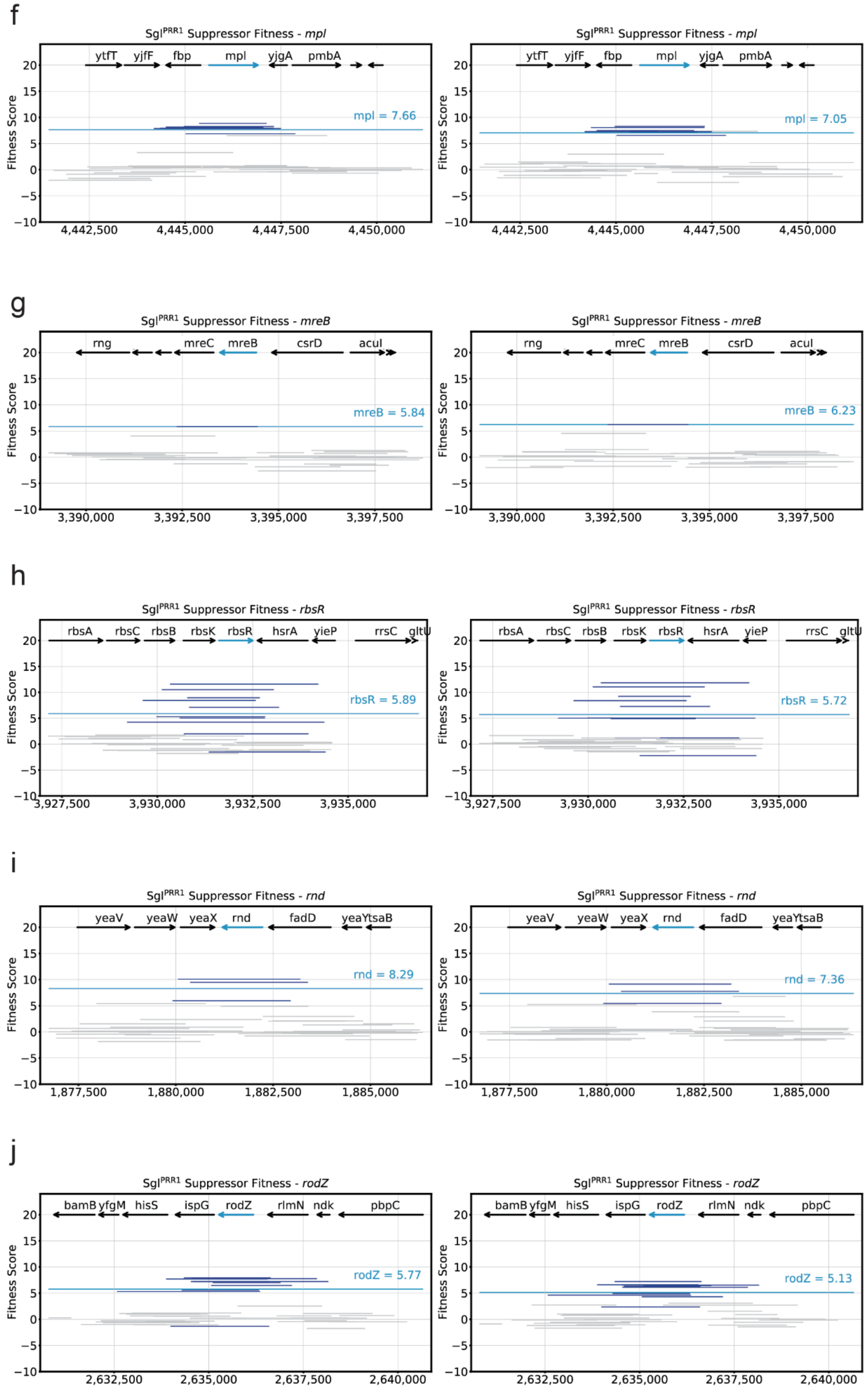
f
SglPRR1 Suppressor Fitness - mpl
ytfT yjfF fbp mpl yjgA pmbA
mpl = 7.66
mpl = 7.05
Fitness Score
4,442,500 4,445,000 4,447,500 4,450,000
g
SglPRR1 Suppressor Fitness - mreB
rng mreC mreB csrD acuI
mreB = 5.84
mreB = 6.23
Fitness Score
3,390,000 3,392,500 3,395,000 3,397,500
h
SglPRR1 Suppressor Fitness - rbsR
rbsA rbsC rbsB rbsK rbsR hsrA yieP rrsC gltU
rbsR = 5.89
rbsR = 5.72
Fitness Score
3,927,500 3,930,000 3,932,500 3,935,000
i
SglPRR1 Suppressor Fitness - rnd
yeaV yeaW yeaX rnd fadD yeaYtsaB
rnd = 8.29
rnd = 7.36
Fitness Score
1,877,500 1,880,000 1,882,500 1,885,000
j
SglPRR1 Suppressor Fitness - rodZ
bamB yfgM hisS ispG rodZ rlmN ndk pbpC
rodZ = 5.77
rodZ = 5.13
Fitness Score
2,632,500 2,635,000 2,637,500 2,640,000

k

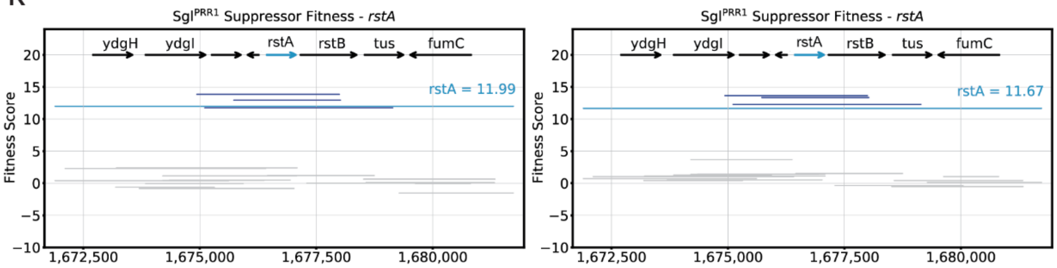
Sgl^{PRR1} Suppressor Fitness - *rstA*
ydgH ydgI rstA rstB tus fumC
rstA = 11.99
Fitness Score
1,672,500 1,675,000 1,677,500 1,680,000
Sgl^{PRR1} Suppressor Fitness - *rstA*
ydgH ydgI rstA rstB tus fumC
rstA = 11.67
Fitness Score
1,672,500 1,675,000 1,677,500 1,680,000

l

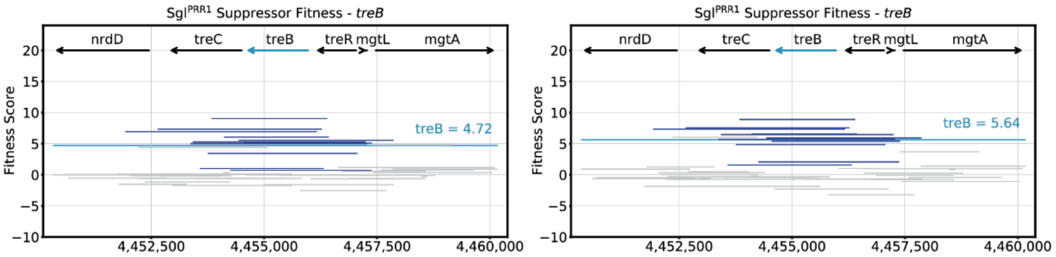
Sgl^{PRR1} Suppressor Fitness - *treB*
nrdD treC treB treR mgtL mgtA
treB = 4.72
Fitness Score
4,452,500 4,455,000 4,457,500 4,460,000
Sgl^{PRR1} Suppressor Fitness - *treB*
nrdD treC treB treR mgtL mgtA
treB = 5.64
Fitness Score
4,452,500 4,455,000 4,457,500 4,460,000

m

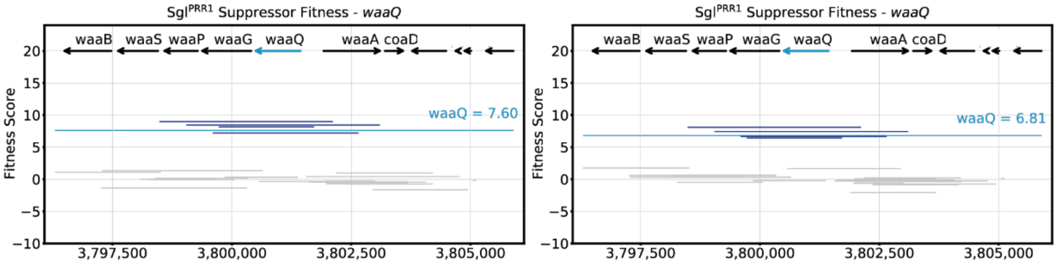
Sgl^{PRR1} Suppressor Fitness - *waaQ*
waaB waaS waaP waaG waaQ waaA coaD
waaQ = 7.60
Fitness Score
3,797,500 3,800,000 3,802,500 3,805,000
Sgl^{PRR1} Suppressor Fitness - *waaQ*
waaB waaS waaP waaG waaQ waaA coaD
waaQ = 6.81
Fitness Score
3,797,500 3,800,000 3,802,500 3,805,000

n

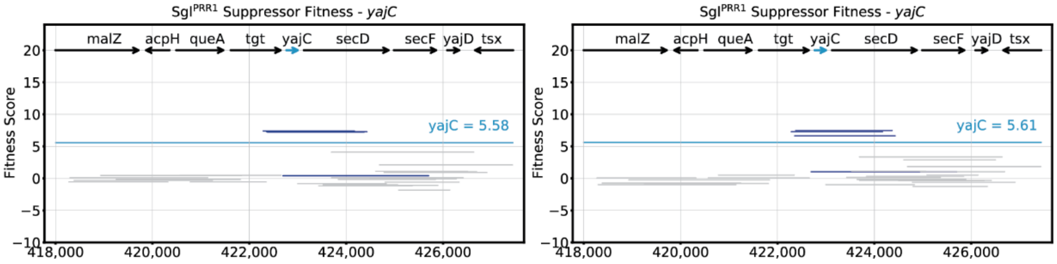
Sgl^{PRR1} Suppressor Fitness - *yajC*
malZ acpH queA tgt yajC secD secF yajD tsx
yajC = 5.58
Fitness Score
418,000 420,000 422,000 424,000 426,000
Sgl^{PRR1} Suppressor Fitness - *yajC*
malZ acpH queA tgt yajC secD secF yajD tsx
yajC = 5.61
Fitness Score
418,000 420,000 422,000 424,000 426,000

o

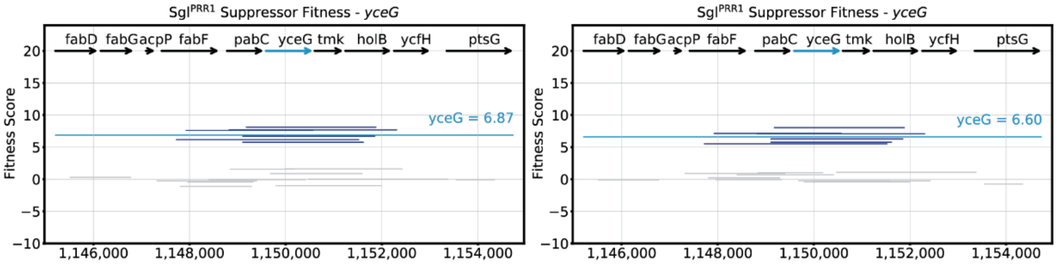
Sgl^{PRR1} Suppressor Fitness - *yceG*
fabD fabGacpP fabF pabC yceG tmk holB ycfH ptsG
yceG = 6.87
Fitness Score
1,146,000 1,148,000 1,150,000 1,152,000 1,154,000
Sgl^{PRR1} Suppressor Fitness - *yceG*
fabD fabGacpP fabF pabC yceG tmk holB ycfH ptsG
yceG = 6.60
Fitness Score
1,146,000 1,148,000 1,150,000 1,152,000 1,154,000

p

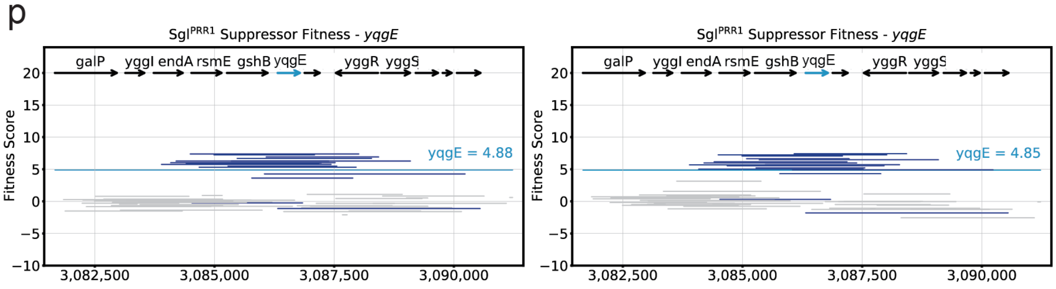

q

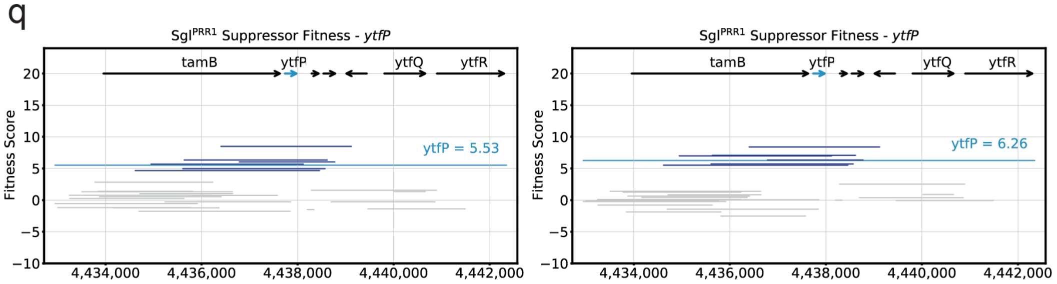

Supplementary Fig. 6: Detailed Dub-seq suppressor screening results against Sgl^{PRR1}. (a-q) Dub-seq plots for suppressor screening against Sgl^{PRR1} zoomed in on a gene of interest with experimental replicates shown side-by-side. Genes of interest are significant hits shown in Fig. 2 or candidate multi-gene multi-copy suppressors. Black arrows represent tracked ORFs during analysis. Teal arrows represent the gene of interest. Dark blue lines represent Dub-seq fragments covering the gene of interest with scores shown as *fscores*. Gray lines represent Dub-seq fragments that do not entirely cover the gene of interest with scores shown as *fscores*. Teal lines represent the *gscore* of the gene of interest.

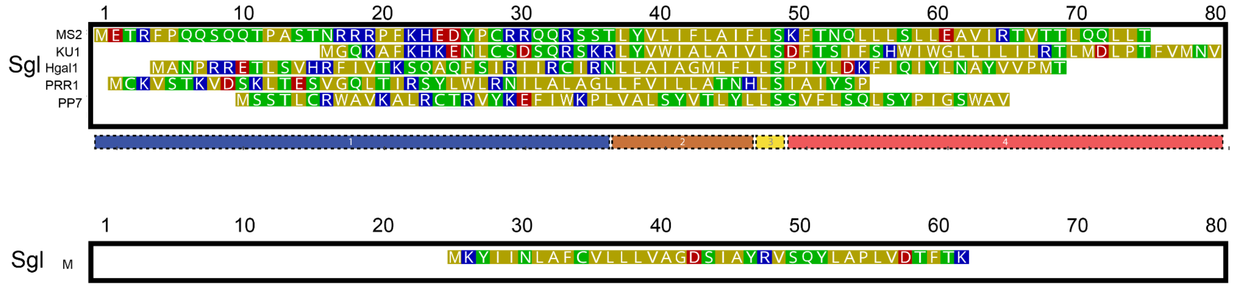

Supplementary Fig. 7: Sequence alignment of *Fiersviridae* lysis proteins. *Fiersviridae* lysis proteins bear little resemblance to each other. The L-like Sgl sequences were centered on the LS motif (Top panel). Domains proposed for L-like Sgl previously (PMID: 28691656) are outlined at the bottom of the diagram. Briefly, domain 1 (Blue) refers to the variable-length, positively charged N-terminus, domain 2 (Brown) refers to the LS-preceding hydrophobic region, domain 3 (yellow) refers to the L-like conserved LS motif, and domain 4 (red) refers to the highly variable C-terminus. For comparison, Sgl^{M} sequence is also shown at the bottom panel.
