# Supplementary material for: Multicopy suppressor screens reveal convergent evolution of single-gene lysis proteins
Source: Nat Chem Biol. 2023 Feb 20;19(6):759–66. doi: 10.1038/s41589-023-01269-7 (PMC10229422; doi:10.1038/s41589-023-01269-7)
Supplement: Supplementary file 2 — Reporting Summary [file 41589_2023_1269_MOESM2_ESM.pdf]

nature portfolio

Corresponding author(s): Vivek K Mutalik, Adam P Arkin

Last updated by author(s): 2022/12/20

Reporting Summary

Nature Portfolio wishes to improve the reproducibility of the work that we publish. This form provides structure for consistency and transparency in reporting. For further information on Nature Portfolio policies, see our Editorial Policies and the Editorial Policy Checklist.

Statistics

For all statistical analyses, confirm that the following items are present in the figure legend, table legend, main text, or Methods section.

n/a	Confirmed	
☐	☒	The exact sample size (*n*) for each experimental group/condition, given as a discrete number and unit of measurement
☐	☒	A statement on whether measurements were taken from distinct samples or whether the same sample was measured repeatedly
☒	☐	The statistical test(s) used AND whether they are one- or two-sided<br>*Only common tests should be described solely by name; describe more complex techniques in the Methods section.*
☐	☒	A description of all covariates tested
☐	☒	A description of any assumptions or corrections, such as tests of normality and adjustment for multiple comparisons
☐	☒	A full description of the statistical parameters including central tendency (e.g. means) or other basic estimates (e.g. regression coefficient) AND variation (e.g. standard deviation) or associated estimates of uncertainty (e.g. confidence intervals)
☒	☐	For null hypothesis testing, the test statistic (e.g. *F*, *t*, *r*) with confidence intervals, effect sizes, degrees of freedom and *P* value noted<br>*Give P values as exact values whenever suitable.*
☒	☐	For Bayesian analysis, information on the choice of priors and Markov chain Monte Carlo settings
☐	☒	For hierarchical and complex designs, identification of the appropriate level for tests and full reporting of outcomes
☒	☐	Estimates of effect sizes (e.g. Cohen's *d*, Pearson's *r*), indicating how they were calculated

Our web collection on statistics for biologists contains articles on many of the points above.

Software and code

Policy information about availability of computer code

Data collection	No specialized software was used to collect the data as part of this study except for the standard Illumina software for calling bases.
Data analysis	All code used in this study is freely available. The following software was used: Python 3.7.3, Dub-seq sequence-processing and analysis scripts can be found on Github as previously reported (PMID 30659179): https://github.com/novichkov-lab/DubSeq (version 1.0). Geneious2022.2.1 was used for plasmid map visualization and locus sequence analysis; The growth curves were plotted using Rstudio Version 1.3.1073 and Inkscape 1.0

For manuscripts utilizing custom algorithms or software that are central to the research but not yet described in published literature, software must be made available to editors and reviewers. We strongly encourage code deposition in a community repository (e.g. GitHub). See the Nature Portfolio guidelines for submitting code & software for further information.

Data

Policy information about availability of data

All manuscripts must include a data availability statement. This statement should provide the following information, where applicable:

- Accession codes, unique identifiers, or web links for publicly available datasets
- A description of any restrictions on data availability
- For clinical datasets or third party data, please ensure that the statement adheres to our policy

Sequencing data have been uploaded to the Sequence Read Archive under BioProject

accession number PRJNA800467 [http://www.ncbi.nlm.nih.gov/bioproject/800467]. Complete data from all experiments (read counts per barcode, fragment scores, and gene scores) are deposited here: [https://doi.org/10.6084/m9.figshare.21714296.v2]. MurJEC structure was downloaded from the Research Collaboratory for Structural Bioinformatics (RCSB) Protein Data Bank, PDB 6CC4. The data underlying Figure 1cd, Figure 2b, Figure 3, Figure 4b and Supplementary Figures 1-6 is provided as source data. There are no restrictions on the availability of the data described in this study

Human research participants

Policy information about studies involving human research participants and Sex and Gender in Research.

Reporting on sex and gender	Not applicable
Population characteristics	Not applicable
Recruitment	Not applicable
Ethics oversight	Not applicable

Note that full information on the approval of the study protocol must also be provided in the manuscript.

Field-specific reporting

Please select the one below that is the best fit for your research. If you are not sure, read the appropriate sections before making your selection.

☒ Life sciences ☐ Behavioural & social sciences ☐ Ecological, evolutionary & environmental sciences

For a reference copy of the document with all sections, see nature.com/documents/nr-reporting-summary-flat.pdf

Life sciences study design

All studies must disclose on these points even when the disclosure is negative.

Sample size	For the barcoded suppressor libraries, we made sure that diversity was sufficiently high to cover most E. coli genes at least once as described in methods. Supplementary Table 1 covers library sizes. For assays using suppressor libraries, experiments were performed in duplicate on different days to ensure reproducibility.<br><br>We performed non-library experiments in biological triplicate (N=3) to facilitate measurements of both mean and dispersion.
Data exclusions	No experimental samples were excluded in this study. Fitness experiments that did not meet pre-established quality criteria and fragments, and genes that did not have sufficient coverage were excluded from all analyses. Also, a few manual analyses were conducted with the preliminary data as detailed in the Methods section
Replication	All library-scale experiments were performed in duplicate on different days. Dub-seq libraries encode gene-level replicates within a library experiment that produce gscores, which are presented in this study.<br><br>All non-library experiments were performed in triplicate on different days. All experiments were successful unless otherwise noted in the manuscript.
Randomization	This study focuses on genetic screens of randomly sheared genomic fragments in presence of a toxic gene (induction of sgl encoded from a plasmid) and looks for suppressors. This study does not involve subjects that require randomization.
Blinding	This study does not involve subjects that require blinding

Reporting for specific materials, systems and methods

We require information from authors about some types of materials, experimental systems and methods used in many studies. Here, indicate whether each material, system or method listed is relevant to your study. If you are not sure if a list item applies to your research, read the appropriate section before selecting a response.

Materials & experimental systems

n/a	Involved in the study
☒	☐ Antibodies
☒	☐ Eukaryotic cell lines
☒	☐ Palaeontology and archaeology
☒	☐ Animals and other organisms
☒	☐ Clinical data
☒	☐ Dual use research of concern

Methods

n/a	Involved in the study
☒	☐ ChIP-seq
☒	☐ Flow cytometry
☒	☐ MRI-based neuroimaging
